# A broadly neutralizing monoclonal antibody induces broad protection against heterogeneous *PRRSV* strains in piglets

**DOI:** 10.1186/s13567-021-00914-0

**Published:** 2021-03-16

**Authors:** Zhigang Zhang, Tianshu Zhai, Mingshuo Li, Kun Zhang, Jingrui Li, Xu Zheng, Chaonan Tian, Rui Chen, Jianhui Dong, En-Min Zhou, Yuchen Nan, Chunyan Wu

**Affiliations:** 1 Department of Preventive Veterinary Medicine, College of Veterinary Medicine, Northwest A&F University, Yangling, Xianyang, Shaanxi China; 2 China Institute of Veterinary Drug Control, Beijing, 100081 China; 3 State Key Laboratory of Plant Physiology and Biochemistry, College of Biological Sciences, China Agricultural University, Beijing, 100193 China; 4 Shaanxi Innolever Biotechnology Co., Ltd., Yangling, Xianyang, 712100 Shaanxi China

**Keywords:** PRRSV, Monoclonal antibody, Broad neutralizing antibodies, Anti-PRRSV therapy, Transcriptome profiling

## Abstract

Neutralizing antibodies (NAbs) have attracted attention as tools for achieving PRRSV control and prevention, but viral antigenic variation undermines the abilities of NAbs elicited by attenuated PRRSV vaccines to confer full protection against heterogeneous PRRSV field isolates. As demonstrated in this study, the monoclonal antibody (mAb) mAb-PN9cx3 exhibited broad-spectrum recognition and neutralizing activities against *PRRSV-1* and *PRRSV-2* strains in vitro. Furthermore, in vivo experiments revealed that the administration of two 10-mg doses of mAb-PN9cx3 before and after the inoculation of piglets with heterologous PRRSV isolates (HP-PRRSV-JXA1 or PRRSV NADC30-like strain HNhx) resulted in significant reduction of the PRRSV-induced pulmonary pathological changes and virus loads in porcine alveolar macrophages (PAMs) compared with the results obtained with mAb-treated isotype controls. Moreover, minimal hilar lymph node PRRSV antigen levels were observed in mAb-PN9cx3-treated piglets. A transcriptome profile analysis of PAMs extracted from lung tissues of piglets belonging to different groups (except for antibody-isotype controls) indicated that mAb-PN9cx3 treatment reversed the PRRSV infection-induced alterations in expression profiles. A gene ontology (GO) enrichment analysis of these genes traced their functions to pathways that included the immune response, inflammatory response, and response to steroid hormone, and their functions in oogenesis and positive regulation of angiogenesis have been implicated in PRRSV pathogenesis. Overall, NADC30-like HNhx infection affected more gene pathways than HP-PRRSV infection. In conclusion, our research describes a novel immunologic approach involving the use of mAbs that confer cross-protection against serious illness resulting from infection with heterogeneous *PRRSV-2* isolates, which is a feat that has not yet been achieved through vaccination. Ultimately, mAb-PN9cx3 will be a powerful addition to our current arsenal for achieving PRRSV prevention and eradication.

**Supplementary Information:**

The online version contains supplementary material available at 10.1186/s13567-021-00914-0.

## Introduction

Porcine reproductive and respiratory syndrome (PRRS) is a viral disease that has economically decimated the swine industry worldwide since its emergence decades ago [1]. PRRS virus (PRRSV), the causative agent of PRRS, is a single-stranded positive-sense RNA virus that belongs to the genus *Betaarterivirus* of the family *Arteriviridae* within the order *Nidovirales* [2–4]. The genome size of PRRSV is approximately 15 kb, and its genome harbours at least 10 open reading frames (ORFs) [5]. The ORF1a and ORF1b sequences comprise approximately 75% of the entire PRRSV genome and encode at least 16 nonstructural proteins involved in the processing of viral polyproteins, genome replication, and transcription, whereas ORFs 2–7 encode eight structural proteins required for virion assembly [5, 6]. Two species of PRRSV exist, namely, *Betaarterivirus suid 1* (formally known as PRRSV-1) and *Betaarterivirus suid 2* (formally known as PRRSV-2), which share approximately 60% nucleotide sequence identity and exhibit serotype differences [4, 7, 8]. However, the overall disease phenotype, gross clinical signs, and genomic organization are similar between the two species [9].

Since the emergence of PRRSV in 1987, a modified live virus (MLV) vaccine of PRRSV has been developed, and this vaccine was made widely available beginning in 1994 with the launch of Ingelvac PRRS® MLV (Boehringer Ingelheim) in the United States. Unfortunately, PRRSV vaccination had only shown limited success due to the constant emergence of new virulent PRRSV variants that have triggered new outbreaks globally. In the late 1990s, an atypical PRRSV strain causing high rates of mortality and abortion in vaccinated herds circulated in the United States [10]. Soon afterward in 2001, circulation of the highly virulent strain MN184 was detected in Canada and Minnesota. This strain was distinct and demonstrated > 14.5% nucleotide dissimilarity with previously characterized *PRRSV-2* isolates [11]. In 2006, a new type of highly pathogenic PRRSV (now referred to as HP-PRRSV), which showed 100% mortality in sows, emerged in South China [12]. Shortly thereafter in 2008, the identification of a new *PRRSV-2* strain, NADC30, was reported in the US, and NADC30-like strains (with mortality rates of 30%–50%) have been isolated in China since 2015 [13].

In general, immunization with modified live virus (MLV) vaccines confers complete protection against challenges with homologous strains. However, MLVs confer only partial or no protection against challenges with genetically heterologous wild-type strains [14, 15], which aligns with the observed emergence of atypical PRRS outbreaks in vaccinated herds [10, 16]. Moreover, after large-scale vaccination with an attenuated vaccine, field isolates obtained during PRRSV outbreaks harbour nucleotide sequences that are nearly identical to those of vaccine strains or sequences resulting from recombination between PRRSV strains circulating in herds and MLV vaccine strains [17–23]. Therefore, a novel strategy is needed to achieve better control of PRRSV.

Analyses of PRRSV-specific antibody kinetics have suggested that the onset of emergence of neutralizing antibodies (NAbs) after infection correlates with virus clearance from the circulation and tissues [24]. Unexpectedly, pig sera from sows exposed to circulating *PRRSV-2* viruses contain broad NAbs with activities against genetically diverse *PRRSV-1* and *PRRSV-2* strains [24, 25], which indicates that unknown PRRSV antigenic epitopes can potentially be shared by diverse PRRSV strains and might evoke the production of broad NAbs against isolates of both PRRSV species.

In our previous work, a panel of PRRSV-specific murine-derived monoclonal antibodies (mAbs) previously raised against baculovirus-expressed PRRSV structural proteins was screened for the detection of PRRSV-infected cells in vitro. One of these mAbs, an IgM-type mAb denoted as mAb-PR5nf1 was characterized, which revealed that the mAb exhibits broad NAb activity against heterogeneous *PRRSV-1* and *PRRSV-2* isolates in both MARC-145 cells and porcine alveolar macrophages (PAMs) in vitro infected with heterologous PRRSV strains [26]. However, because mAb-PR5nf1 is an IgM antibody, its in vivo applicability is expected to be limited due to the inability of this type of antibodies to penetrate tissue.

In the present study, the novel mAb mAb-PN9cx3 (IgG), which showed broad-spectrum recognition of heterogeneous PRRSV strains, was investigated to assess its neutralization activity in PAMs against both *PRRSV-1* and *PRRSV-2* isolates. Subsequent in vivo protection experiments demonstrated that the administration of 20 mg of mAb-PN9cx3 (two 10-mg doses before and after the inoculation of piglets with heterogeneous HP-PRRSV-JXA1 or NADC30-like HNhx) significantly alleviated the clinical signs of PRRS and decreased the virus loads in PAMs and hilar lymph nodes of PRRSV-inoculated piglets. Our research revealed a novel immunologic approach that can generated broad-spectrum protection against heterologous PRRSV, which is a feat that not yet been achieved through normal MLV vaccination.

## Materials and methods

### Cells and viruses

MARC-145 cells were cultured in Dulbecco’s modified Eagle’s medium (Biological Industries, Israel) supplemented with 10% foetal bovine serum (Biological Industries), 100 U/mL penicillin, and 100 mg/mL streptomycin and were incubated at 37 °C with 5% CO_2_. Porcine alveolar macrophages (PAMs) were collected from specific-pathogen-free pigs and maintained in RPMI 1640 medium (Biological Industries) supplemented with 10% FBS. The PRRSV strains used in this study included SD16 (GenBank: JX087437.1), JXA1 (GenBank: EF112445.1), GD-HD (GenBank: KP793736.1), VR2332 (GenBank: EF536003.1), NADC30-like Chinese isolate HNhx (GenBank: KX766379) and *PRRSV-1* Chinese isolate GZ11-G1 (GenBank: KF001144.1). All PRRSV strains were propagated and titrated in MARC-145 cells.

### Monoclonal antibody production and purification

The parental hybridoma clone secreting PRRSV-specific mAb-PN9cx3 was generated as previously described [27]. Briefly, mice were immunized with SF9 cells infected with recombinant baculovirus expressing GP3 protein of strain VR2385 and then subjected to hybridoma fusion based on standard fusion protocols. The supernatants of the surviving hybridomas were screened using immunofluorescence assays (IFAs) to detect the immunofluorescence of PRRSV VR2385-infected CRL-11171 cells (a parallel subcloned cell line derived from MA104). Parental hybridoma cell clones were propagated via three additional rounds of subcloning to obtain the mAb-PN9cx3-producing clone. mAb-PN9cx3 was purified from mouse ascites using a HiTrap Protein G HP column (GE Healthcare) in accordance with the manufacturer’s instructions. Mouse ascites containing mAb-PN9cx3 were generated by Genscript Co., Ltd., in BALB/c mice (Nanjing, Jiangsu, China). The purified mAb was eluted with 0.1 M glycine (pH 2.7), dialyzed against phosphate-buffered saline (PBS) overnight at 4 °C, concentrated using 100-kDa cutoff ultrafiltration centrifugal tubes (EMD Millipore) and quantified using a BCA protein quantification kit (Thermo Fisher Scientific). The previously described mAb-2G8 (IgG) against HEV-ORF2 protein was used as an isotype control and was purified using the same methods used to purify mAb-PN9cx3 [28].

### Immunofluorescence assay (IFA)

MARC-145 cells were seeded into 24-well plates and infected with different PRRSV strains at an MOI of 1. Twenty-four hours post-infection (hpi), the cells were fixed in 4% paraformaldehyde (Sigma-Aldrich) and permeabilized with PBS containing 0.5% Triton X-100 (Sigma-Aldrich). IFAs were performed using mAb-PN9cx3 and anti-PRRSV convalescent swine serum obtained from a PRRSV-SD16-infected pig (animal #345) as described previously [29]. Specific interactions were detected using Alexa Fluor®555-labelled goat anti-mouse secondary antibody (Thermo Fisher Scientific) and FITC-labelled goat anti-swine secondary antibody (Jackson ImmunoResearch). The cell nuclei were visualized using DAPI (Thermo Fisher Scientific), and the IFA samples were observed under a Leica DM1000 fluorescence microscope (Leica, Germany). All the images were captured and processed using Leica Application Suite X (Version 1.0. Leica Microsystems).

### Single-replication virus-neutralization assay of PAMs

To analyse the mAb-PN9cx3-mediated inhibition of PRRSV infection, an in vitro PRRSV-specific inhibition assay was conducted using PAMs as previously described with modifications [26, 30]. Briefly, PAMs were seeded into 24-well plates at a density of 1 × 10^6^ cells/well, and the plates were then incubated at 37 °C for 3 h. mAb-PN9cx3 at the indicated dose was incubated with various PRRSV strains (10^5^ TCID_50_) at 37 °C for 1 h. The mAb-virus mixtures were then added to PAMs, and the PAMs were then incubated for 1 h at 37 °C. The supernatants were subsequently replaced with fresh medium, and the PAMs were incubated at 37 °C for an additional 24 h and then harvested for subsequent experiments. Cell culture supernatants containing progeny virus were titrated in MARC-145 cells to evaluate the production of infectious virions.

### Sandwich ELISA-based PRRSV virion capture assay

A sandwich ELISA-based PRRSV virion capture assay was conducted as previously described [26]. Briefly, the wells of 96-well polystyrene microplates (Corning, Corning, NY, USA) were coated with 400 ng of mAb-PN9cx3 in 100 μL of PBS (pH 8.0) per well. The plates were incubated overnight at 4 °C, and each well was then blocked with 2.5% gelatine in PBS supplemented with 0.5% Tween 20 (Sigma-Aldrich). Subsequently, HP-PRRSV-SD16 (100 μL, 10^6.5^ TCID_50_/mL) was added to the wells, and the plates were incubated for 1 h at 37 °C. For the isotype control, all the steps were performed using mAb-2G8 at the same amount as mAb-PN9cx3. Anti-PRRSV convalescent pig serum (1:200 dilution in PBS) was used to detect the captured PRRSV virions. The binding of pig antibodies was revealed using HRP-conjugated goat anti-pig IgG (Jackson ImmunoResearch, West Grove, PA, USA) and 3,3′,5,5′-tetramethylbenzidine (TMB) substrate (TianGen Biotech, Beijing, China). The absorbances of the individual wells were measured using a Victor^TM^X5 Multilabel Plate Reader (PerkinElmer, Waltham, MA, USA) at a wavelength of 450 nm.

### PRRSV virion attachment assay

A PRRSV virion attachment assay was conducted as previously described with modifications [31]. Briefly, 1 × 10^6^ PAMs were seeded into each well of 24-well plates, and the plates were then incubated for 3 h. The virus (PRRSV-SD16 strain, 10^5^ TCID_50_, 0.1 MOI) was mixed with 1 μM mAb-PN9cx3, and the mixture was then incubated for 1 h at 37 °C and cooled at 4 °C for 30 min prior to their addition to PAMs. Subsequently, prechilled PAMs were coincubated with the cooled mixture of the PRRSV-SD16 virus and mAb-PN9cx3 and incubated at 4 °C for 1 h; in parallel, prechilled PAMs were incubated with an equal dose of chilled HP-PRRSV-SD16 virus alone. During these incubations, the mixtures were maintained cool to avoid triggering endocytosis. Subsequently, the treated PAMs were washed three times with cold PBS to remove unbound virions, harvested and analysed by RT-qPCR assays to evaluate the viral RNA levels reflecting numbers of virions that had specifically bound to the surfaces of PAMs.

### Western blot analysis

Sodium dodecyl sulphate–polyacrylamide gel electrophoresis (SDS-PAGE) and Western blot analysis were conducted as previously described [32]. Briefly, PAMs were harvested using 1 × Laemmli sample buffer (Bio-Rad Laboratories, Hercules, CA, USA), and the samples were then denatured at 98 °C for 10 min, transferred to ice for another 5 min, and subjected to SDS-PAGE. An equal amount of protein sample was loaded per lane, and the proteins were separated using 12% SDS-PAGE gels and then transferred to PVDF membranes (Millipore, Billerica, MA, USA). The membranes were blocked with 5% skim milk and probed with homemade mAb-6D10 against PRRSV-N protein and anti-β-tubulin mAb (Abcam, Cambridge, MA, USA). The specific binding between antibodies and their corresponding targets was detected using HRP-conjugated goat anti-mouse IgG (Jackson ImmunoResearch) and visualized using ECL substrate (Bio-Rad Laboratories). The chemiluminescence signal was digitally recorded using a ChemiDoc™ MP Imaging System (Bio-Rad Laboratories) and then analysed using Image Lab software (Version 5.1, Bio-Rad Laboratories).

### RNA isolation and quantitative real-time PCR (qPCR)

Total RNA from PAMs was extracted using the TRIzol reagent (Invitrogen, Grand Island, NY, USA) according to the manufacturer’s instructions. Reverse transcription and qPCR were conducted using a PrimeScript RT reagent Kit (TaKaRa, Dalian, China) as previously described [26]. Transcripts of GAPDH were also amplified and used to normalize the total RNA input. The relative expression levels of the target genes were quantified using the 2^−ΔΔ^ Ct method. For the in vivo assessment of viremia and the virus loads in PAMs, serum samples and PAMs from piglets were harvested using the TRIzol reagent. Reverse transcription and qPCR were conducted as described above using PRRSV N-specific primers. For the absolute quantification of viral RNA copies, a dilution series of pet28a-PRRSV-N plasmids was used to generate a standard curve for calculating the number of RNA copies. All sequences of primers used for qPCR are listed in Table 1.

**Table 1:** **List of primers used in this study.**

Genes	Forward (5′–3′)	Reverse (5′–3′)
*PRRSV-1-ORF7*	ATGGCCGGTAAAAATCAGAGCC	TTAATTCGCACCCTGACTGG
*PRRSV-2-ORF7*	ATGCCAAATAACAACGGCAAGCAGC	TCATGCTGAGGGTGATGCTGTG
*GAPDH*	CCTTCCGTGTCCCTACTGCCAAC	GACGCCTGCTTCACCACCTTCT

### Ethics statement and animal experiments

Four-week-old piglets (21 in total) were obtained from a PRRSV-free pig farm near Yangling, Shaanxi, and further subjected to screenings for infection with classic swine fever virus, PRRSV, porcine circovirus 2, and African swine fever virus (ASFV) as well as screenings for corresponding antibodies by a government-authorized agency (Shaanxi Innolever Biotechnology Co., Ltd., Yangling, Shaanxi, China). Only piglets that were negative for all examined pathogens and for PRRSV- and ASFV-specific antibodies were selected for the experiments. The animal protocol outlining the PRRSV challenge experimental procedures was reviewed and approved by the Animal Welfare Committee of Northwest A&F University. The animals were randomly divided into seven groups (*n* = 3), and each group was maintained in a separate isolation room. Among the seven groups, two groups received inoculations with HP-PRRSV-JXA1 or NADC30-like HNhx, two groups were treated with mAb-PN9cx3 2 days prior to challenge with HP-PRRSV-JXA1 or NADC30-like HNhx, two groups received isotype mAb (2G8) treatment 2 days prior to challenge with the two abovementioned PRRSV isolates, and the remaining group was included as a negative control (MOCK) group. Details of the piglet groupings are provided in Table 2.

**Table 2:** **Animal groups and corresponding treatments.**

Group name	Injected mAb	Challenge virus
MOCK	PBS	PBS
JXA1	PBS	PRRSV-JXA1
HNhx	PBS	PRRSV-NADC30 like HNhx
JXA1/mAb-2G8	mAb-2G8	PRRSV-JXA1
HNhx/mAb-2G8	mAb-2G8	PRRSV- NADC30 like HNhx
JXA1/mAb-PN9cx3	mAb-PN9cx3	PRRSV-JXA1
HNhx/mAb-PN9cx3	mAb-PN9cx3	PRRSV- NADC30 like HNhx

At minus 2 days (−2 days) post-inoculation (dpi), 10 mg of mAb-PN9cx3 or mAb-2G8 (2 mg/mL in PBS) was administered intravenously (IV) via the lateral auricular vein of each piglet in the corresponding mAb-treated groups. The same volume of PBS was administered at −2 dpi to the piglets in all the PRRSV challenge-only groups using the same injection route. At 0 dpi, all the piglets except those belonging to the negative control group were challenged with 2 mL of HP-PRRSV-JXA1 or NADC30-like HNhx virus stock (10^5^ TCID_50_/mL) via both intranasal and intramuscular administration (1 mL of virus stock per administration route), whereas the piglets in the MOCK group (negative controls) received the same volume of PBS. One day later (1 dpi), an additional 10-mg dose of mAb-PN9cx3 or mAb-2G8 was administered via the auricular vein to the mAb-treated groups. All the animals were euthanized and necropsied at 21 dpi. A schematic illustration of the entire animal experimental protocol is provided in Figure 5. Serum samples were collected from all the piglets at 0, 7, 14, and 21 dpi and evaluated using an IDEXX HerdChek PRRS X3 ELISA kit (IDEXX, Westbrook, ME, USA) for seroconversion. Viremia was determined by absolute quantification using the qPCR-based method described above.

To evaluate the protection efficiency of mAb-PN9cx3 against PRRSV in piglets, the lungs of the piglets were examined for gross pathological changes immediately after autopsy at 21 dpi. A previously described scoring system was used for the assessment of gross lung lesions [33]. Briefly, each lung lobe (including the anterior, middle, and caudal parts from the ventral and dorsal aspects and accessory lobes) was separately assigned a number of points (100 points total). According to the pathological changes observed in each part of each lobe, each piglet was assigned a score to reflect the percentage of the entire lung exhibiting gross visible signs of pneumonia. Lung tissues from the piglets were sampled for histopathologic examination. All tissue samples were fixed in 10% neutral buffered formalin and embedded in paraffin blocks using routinely established methods. The blocks were sectioned, and the sections were analysed using routine histological procedures after staining with haematoxylin and eosin (H&E) to facilitate visual detection of the micropathological changes.

To examine the replication of PRRSV in pulmonary hilar lymph nodes (LNs), hilar LNs were collected at necropsy, embedded in optimal cutting temperature (OCT) compound in cryomoulds and stored at −80 °C before use. Cryostat Sections (5-μm-thick) were then mounted onto gelatine-coated slides, fixed with ice-cold acetone, and air-dried. After washing with PBS-Tween 20, the sections were blocked with swine anti-PRRSV antibody-negative serum and examined for the presence of PRRSV antigen using anti-PRRSV nucleocapsid (N) monoclonal antibody (mAb-6D10). The presence of specific antibody-protein binding was then detected using FITC-labelled goat anti-mouse secondary antibody (Thermo Fisher Scientific). The slides were counterstained with SlowFade Gold Antifade reagent containing DAPI (Thermo Fisher Scientific) and observed using a Leica DM1000 fluorescence microscope.

### Enzyme-linked immunosorbent assay (ELISA)

For the evaluation of murine IgG in pig serum after the intravenous injection of mAb-PN9cx3 and mAb-2G8, serum samples from the indicated groups of piglets were subjected to an ELISA of mouse IgG using a Mouse IgG Quantification ELISA Kit (BioVision, Milpitas, CA, USA) according to the manufacturer’s instructions with modifications. Briefly, the serum of piglets belonging to the MOCK group that were not injected with mAbs was diluted in PBS at a 1:5 ratio, and the indicated amount of mAb-PN9cx3 was then added to the PBS-diluted pig serum for standard curve calculation. The serum samples from the experimental groups were diluted in PBS at a 1:5 ratio for mouse IgG quantification.

For evaluation of the anti-antibody response (AAR) in mAb-treated piglets, 96-well polystyrene microplates (Corning Inc., Corning, NY, USA) were coated with 200 ng of mAb-PN9cx3 or mAb-2G8 in a volume of 100 μL of PBS (pH 8.0) overnight at 4 °C. The plates were further blocked with 5% skim milk in PBS containing 0.5% Tween 20 (Sigma-Aldrich). Diluted serum samples (1- to 256-fold dilution in PBS) were added to the wells, and the plates were incubated for 1 h at 37 °C and washed with PBS containing 0.5% Triton X-100 (Sigma-Aldrich). The binding of antibodies to the coated mAbs was detected using HRP-conjugated goat anti-pig IgG (Jackson ImmunoResearch) and visualized with a TMB substrate kit (Tiangen Biotech). The absorbance of each well at 450 nm was measured using a Victor ™ X5 Multilabel Plate Reader (Perkin Elmer).

### MARC-145 cell-based virus neutralization assay

A virus neutralization assay using MARC-145 cells was performed to distinguish the PRRSV-neutralizing mAbs as previously described with modifications [34, 35]. Briefly, PRRSV-PRRSV-SD16 was used as the target virus in the assay, and an MOI of 0.1 was used in each assay. The test samples (mAb-PN9cx3 and mAb-2G8 at different doses) were diluted with DMEM, incubated with the virus for 1 h at 37 ℃ and then used for the inoculation of MARC-145 cells. An IFA with the N-specific mAb-PP7EF11 was conducted 18 h after inoculation, and the florescence spots were calculated and compared to determine the virus neutralizing activity of the test samples. The reciprocal of the antibody dose that reduced the replication of PRRSV (as determined by florescence focus) by 50% compared with that observed in the no-mAb control group was counted as the virus neutralization dose.

### Transcriptome analysis

Bronchoalveolar lavage fluid (BALF) samples were collected via lung lavage with sterile PBS from the piglets (*n* = 3) experimentally infected with PRRSV with or without mAb treatments. PAMs from the BALF samples were harvested by centrifugation at 300× *g* for 10 min, and the viability of PAMs was evaluated after the cells were stained with trypan blue. A total of 1 × 10^7^ viable PAMs were harvested using 1 mL of the TRIzol reagent (Thermo Fisher Scientific) according to the manufacturer’s protocol. RNA extraction, cDNA library construction, and RNA sequencing from TRIzol-harvested PAMs were conducted by GENEWIZ Co., Ltd. (Suzhou, Jiangsu, China). RNA-seq libraries were generated using the Illumina system (RS-122-2201) using the manufacturer’s protocols. The reference pig genome assembly (*Sus scrofa* v11.1) was downloaded from Ensemble websites [36]. The transcript abundances were converted to transcripts per million (TPM) units using Kallisto, and the TPM value for a given gene was defined using the most abundant transcript associated with that particular gene. Differential gene expression analysis was performed using sleuth_0.30.0 [37]. Differentially expressed genes were filtered using the following parameters: TPM ≥ 1, fold-change (FC) ≥ 2, and q-value < 0.05. Heatmaps were drawn using the R package tool “pheatmap” from The R Project for Statistical Computing [38]. Functional enrichments were performed using DAVID 6.8. The RNA-seq datasets generated in this study can be found in the aforementioned NCBI Gene Expression Omnibus (GEO Accession no. GSE156504).

### Statistical analysis

The statistical analyses were performed using GraphPad Prism version 5.0 (GraphPad Software, San Diego, CA, USA). The statistical significance of the differences between two groups and among more than two groups was determined by Student’s *t*-test and one-way analysis of variance (ANOVA), respectively. *P* < 0.05 was considered statistically significant.

## Results

### mAb-PN9cx3 recognizes heterogeneous PRRSV isolates of both *PRRSV-1* and *PRRSV-2*

**Figure 1:**
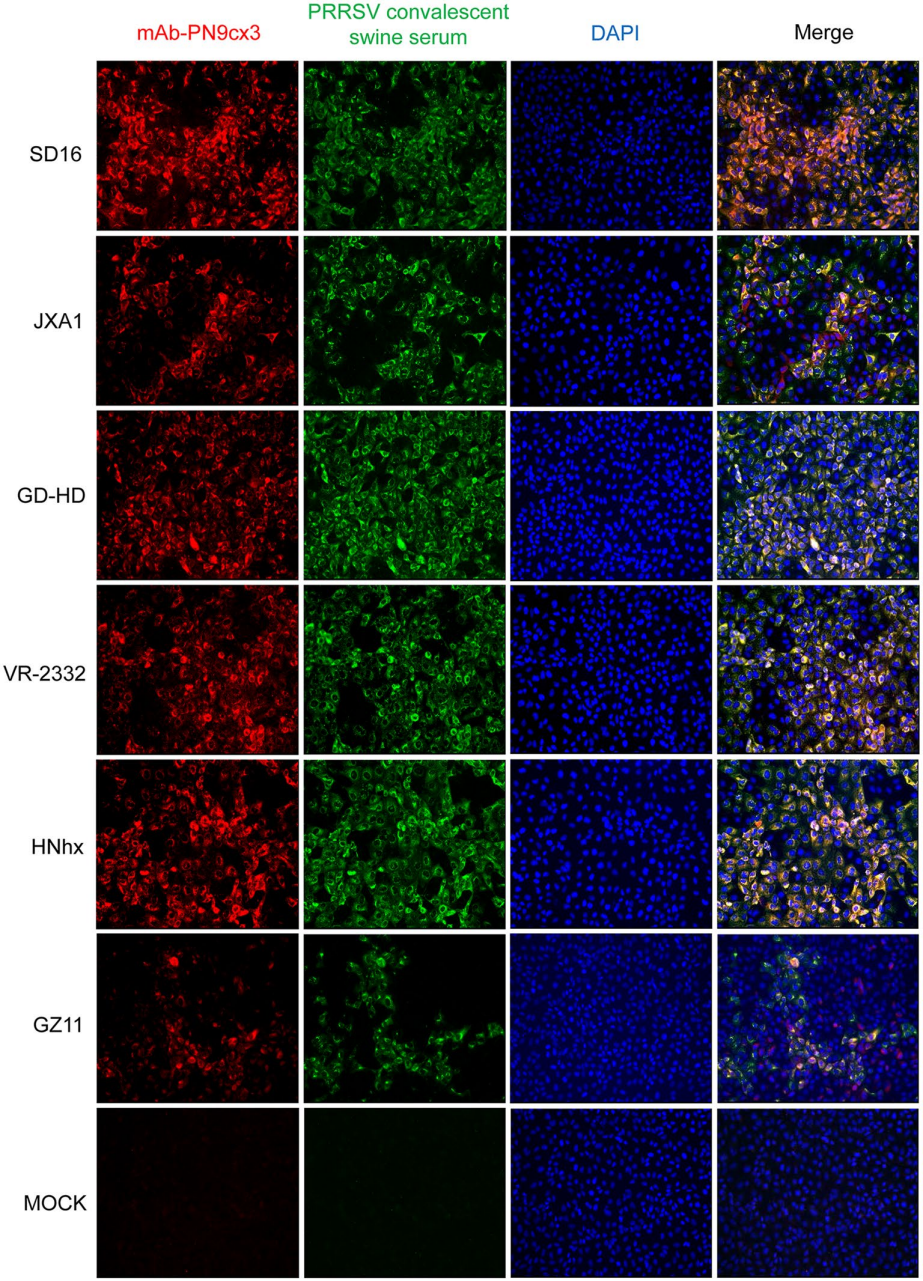
**mAb-PN9cx3 recognizes heterogeneous isolates of both PRRSV-1 and PRRSV-2 species in immunofluorescence assays**. MARC-145 cells were infected with PRRSVs at an MOI of 1 for 24 h, fixed in 4% paraformaldehyde and permeabilized with 0.5% Triton X-100 prior to incubation with a cocktail containing mAb-PN9cx3 hybridoma supernatant and PBS-diluted PRRSV convalescent swine serum (1:200 dilution). After a 1-h incubation, the interactions between the primary antibodies and targets were revealed by the binding of Alexa Fluor®555-labelled goat anti-mouse IgG (H + L) (red) and FITC-labelled goat anti-swine IgG (green). Uninfected MARC-145 cells (MOCK) served as a negative control. The PRRSV strains used for infection included three HP-PRRSV isolates (SD16, Accession No. JX087437.1; JXA1, GenBank Accession No. EF112445.1; and GD-HD, Accession No. KP793736.1), a *PRRSV-2* prototype strain (VR2332, GenBank Accession No. EF536003.1), the NADC30-like Chinese isolate HNhx (GenBank: KX766379), and a Chinese *PRRSV-1* isolate (GZ11, GenBank Accession No. KF001144.1).

The parental clone of mAb-PN9cx3 (Clone No. PP3aC1) was developed two decades ago via the immunization of mice with whole insect cells infected with recombinant baculovirus expressing GP3 of VR2385 strain (not purified GP3) followed by IFA screening of hybridoma supernatant using PRRSV-VR2385-infected CRL11171 cells [39]. IFA and fixed-cell ELISAs revealed that an antibody produced by the original mAb-PP3aC1 clone recognized VR2385-infected CRL11171 cells but not detergent-harvested cell lysates of VR2385-infected CRL11171 cells [39]. After several rounds of subcloning, mAb-PN9cx3 was generated. To further assess the reactivity spectrum of mAb-PN9cx3 towards diverse PRRSV strains, we collected representative strains from both *PRRSV-1* and *PRRSV-2* species, including the classical PRRSV strain VR2332, the highly pathogenic PRRSV strains SD16 and JXA1, GD-HD, NADC30-like Chinese isolate HNhx, and the *PRRSV-1* Chinese isolate GZ11. Based on our IFA results, all PRRSV strains tested in this study showed similar binding reactivities to mAb-PN9cx3, whereas the mAb did not react with uninfected MARC-145 cells (Figure 1). Moreover, PRRS convalescent serum containing polyclonal antibodies from PRRSV-infected pigs was used as a positive control to confirm the replication of PRRSV in MARC-145 cells. These results demonstrated that mAb-PN9cx3 exhibits broad-spectrum binding reactivity with heterogeneous isolates across two PRRSV species.

### mAb-PN9cx3 acts as a neutralizing antibody against PRRSV infection of PAMs

**Figure 2:**
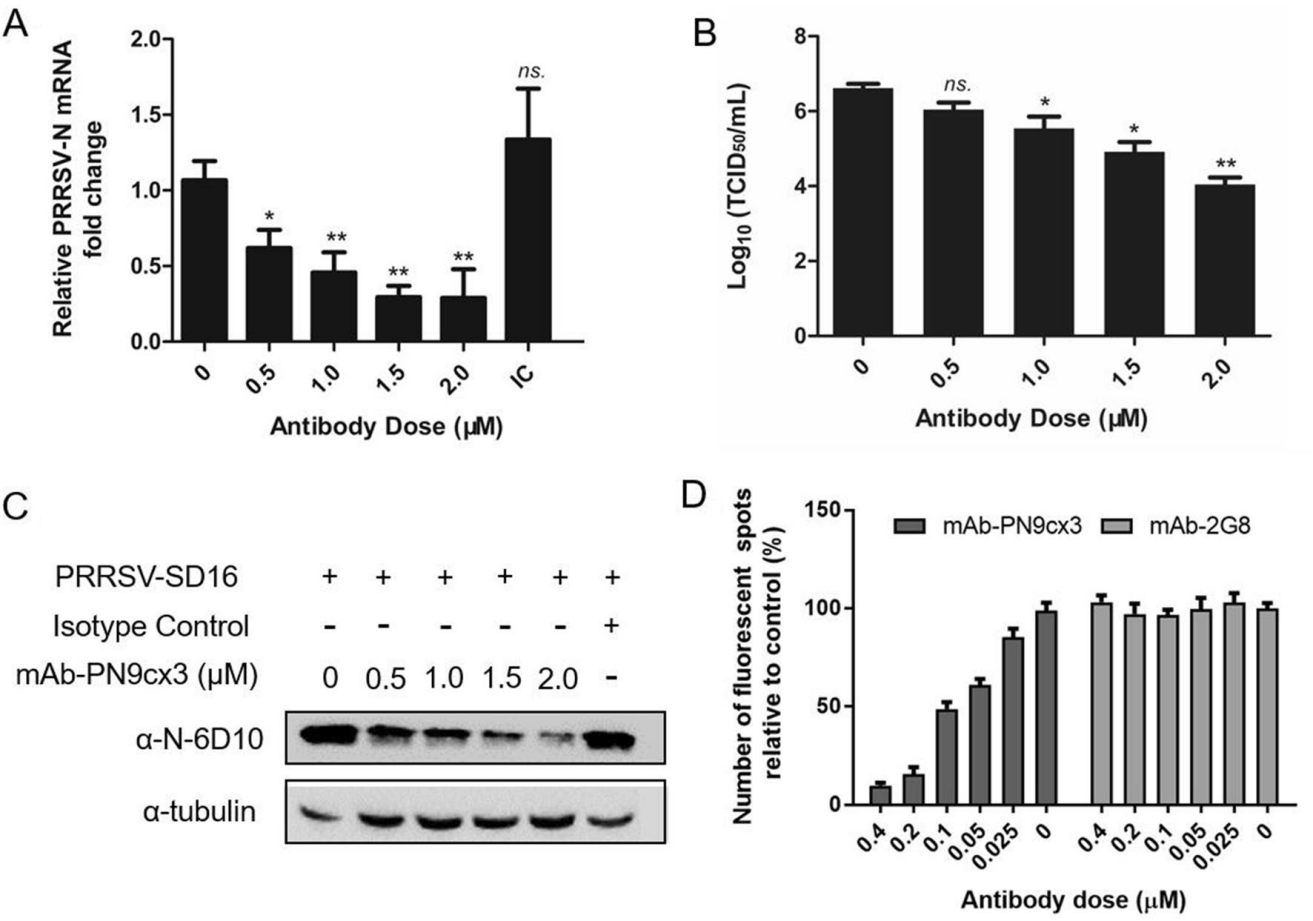
**mAb-PN9cx3 neutralizes HP-PRRSV-SD16 infectivity in primary PAMs**. **A** PRRSV-N protein transcript levels detected by RT-qPCR. PAMs were infected with PRRSV-SD16 virus at an MOI of 0.1 or PRRSV-SD16 virus pretreated (1-h incubation time) with mAb-PN9cx3 at different concentrations or with mAb-2G8 (isotype control, IC) at a concentration of 2 μM. After inoculation with the mAb-virus mixtures, the PAMs were incubated for 1 h, and the unbound PRRSV virions were removed by washing the cells with fresh medium. The replication of PRRSV at 24 h post-infection was determined by RT-qPCR analysis of the PRRSV-N mRNA transcripts. The experiment was repeated three times. The significant differences between the mAb-PN9cx3-treated PRRSV-SD16-challenged group and the untreated PRRSV-SD16-infected group are marked by asterisks: * (*p* < 0.05) and ** (*p* < 0.01). **B** Virus yields in culture supernatants of PAMs infected with the PRRSV-SD16 strain after preincubation with mAb-PN9cx3 (from 0 to 2 μM). Supernatants were collected at 24 hpi and titrated in MARC-145 cells. The significant differences between the 0 μM mAb-PN9cx3-treated group and the indicated groups are marked by asterisks: * (*p* < 0.05) and ** (*p* < 0.01). **C** Western blotting of PAMs infected with PRRSV-SD16 virus at an MOI of 0.1 or PRRSV-SD16 virus pretreated (1-h incubation time) with mAb-PN9cx3 at the indicated concentrations or mAb-2G8 (isotype control, IC) at a concentration of 2 μM. After inoculation with the mAb-virus mixture, the PAMs were incubated for 1 h, and the unbound PRRSV virions were removed by the washing cells with fresh medium. The PAMs were then incubated for 24 h at 37 °C prior to lysis for SDS-PAGE and Western blotting to determine the PRRSV N protein levels. mAb-2G8 was included as an antibody-isotype control. **D** PRRSV-SD16 was used as the target virus in the assay at an MOI of 0.1 and incubated with mAb-PN9cx3 and mAb-2G8 at different doses for 1 h at 37 ℃ prior to the inoculation of MARC-145 cells. An IFA with the PRRSV-N-specific mAb-PP7EF11 was conducted 18 h after inoculation, and the florescence spots were counted and compared with those found in the no-mAb control group.

Because mAb-PN9cx3 was found to broadly recognize PRRSV isolates of both *PRRSV-1* and *PRRSV-2*, we aimed to determine whether this mAb could broadly neutralize PRRSV infectivity. In our previous research, similar PRRSV-neutralizing activity was observed with a mAb against virus infection in MARC-145 and PAMs [26]. Because PAMs are the primary target of PRRSV infection in vivo and are permissive for PRRSV infection in vitro, we used cultured PAMs for infectivity experiments. However, cultured PAMs comprise a heterogeneous group of macrophages that do not form a single cell layer in culture. This obstacle prompted us to directly test the viral neutralization ability of mAb-PN9cx3 and virus replication by measuring the intracellular PRRSV-N protein and mRNA levels in PAMs and the numbers of progeny viruses in culture supernatants as previously described [26, 30]. Using the indicated doses of mAb-PN9cx3, we first incubated the mAb with the PRRSV-SD16 strain (10^5^ TCID_50_). A preincubated mAb-PN9cx3-virus mixture was then added to PAMs to evaluate the efficiency of virus neutralization via measurements of the intracellular viral RNA levels by qPCR. The results demonstrated a significant decrease in the PRRSV-N mRNA levels after preincubation of the virus with a minimal concentration of mAb-PN9cx3 (0.5 μM) relative to the results obtained with the incubation of the virus with the isotype control (2 μM mAb-2G8) and the negative control (0 μM mAb-PN9cx3) (Figure 2A). Moreover, an incremental reduction in the PRRSV-N mRNA levels was observed with increases in the mAb-PN9cx3 doses; as the mAb concentration increased to 1.5 μM or 2.0 μM, the PRRSV-N mRNA level decreased by 60%-70% (Figure 2A). Moreover, the evaluation of progeny PRRSV in PAM culture supernatants revealed significant differences among groups, and a nearly two-log10 reduction in the numbers of infectious virions was found in the supernatants of the 2 μM mAb-PN9cx3-treated group compared with those of the PRRSV-inoculated PAMs without mAb pretreatment (Figure 2B). Importantly, these trends were consistent with the finding that the PRRSV-N mRNA levels decreased with increases in the amounts of mAb-PN9cx3 (Figure 2B). Moreover, a Western blot analysis of the intracellular PRRSV-N protein levels in PRRSV-infected PAMs showed that preincubation of the virus with mAb-PN9cx3 at a concentration as low as 0.5 μM reduced the N protein levels, which suggested that PRRSV replication was inhibited. This inhibitory effect became increasingly stronger with the addition of increasing concentrations of mAb-PN9cx3; moreover, the isotype control exerted a minimal inhibitory effect on PRRSV infectivity (Figure 2C). In addition, the neutralizing activity of mAb-PN9cx3 was confirmed by the observation of reduced florescence spots of mAb-PN9cx3-incubated virus in MARC-145 cells (Figure 2D). Collectively, these data suggest that mAb-PN9cx3 is a PRRSV-specific neutralizing monoclonal antibody that efficiently inhibits PRRSV infection in both PAMs and MARC-145 cells.

### mAb-PN9cx3 is a broad neutralizing mAb that blocks infection with heterogeneous *PRRSV-1* and *PRRSV-2* isolates

**Figure 3:**
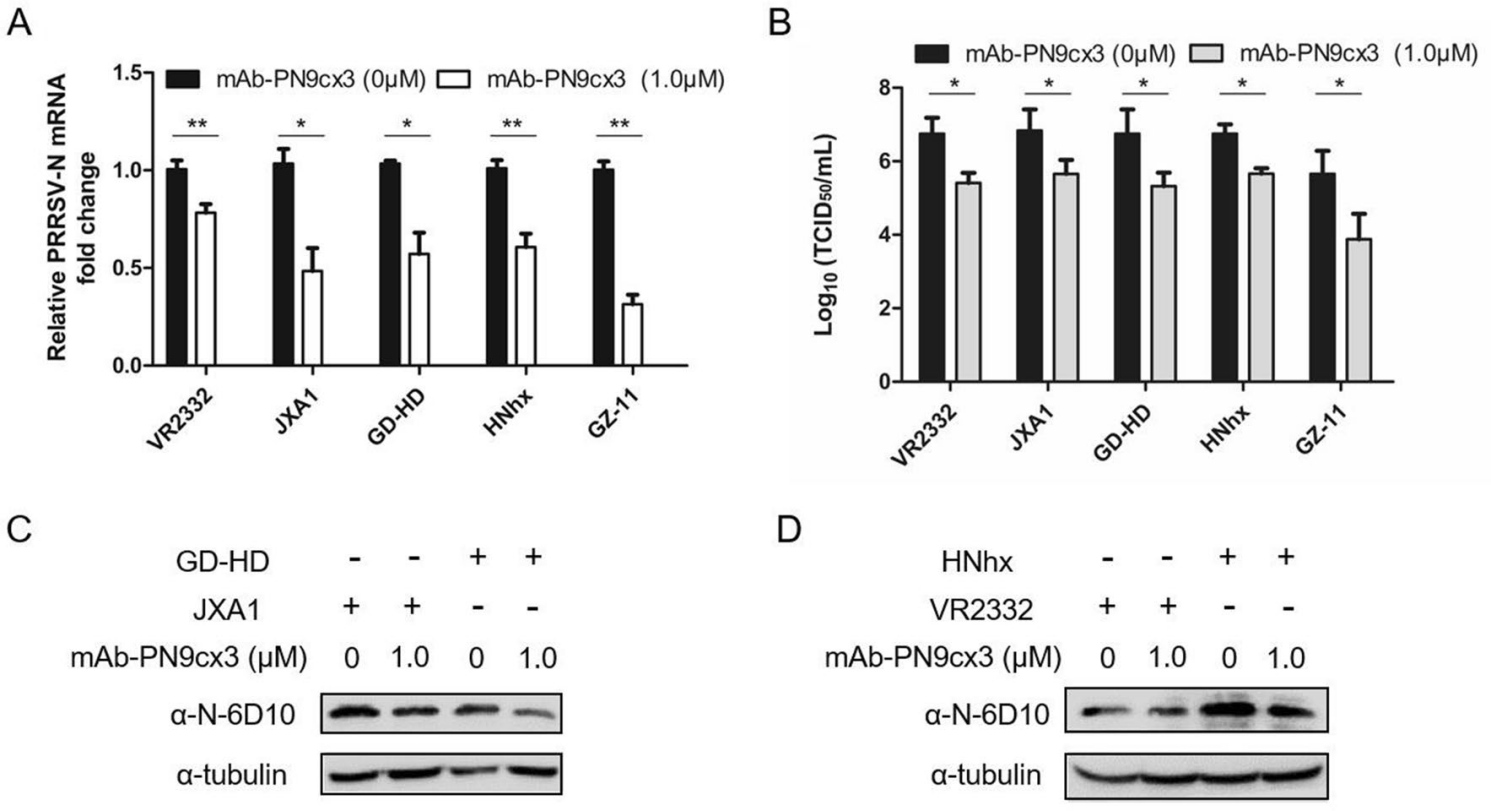
**mAb-PN9cx3 is a broad neutralizing antibody against strains of both PRRSV-1 and PRRSV-2**. **A** The PRRSV N transcript levels detected by RT-qPCR. PAMs were infected with PRRSV-SD16 virus at an MOI of 0.1 or PRRSV-SD16 virus pretreated (1-h incubation time) with 1 μM mAb-PN9cx3. After inoculation with the mAb-virus mixture, the PAMs were incubated for 1 h, and the unbound PRRSV virions were removed by washing the cells with fresh medium. The replication of PRRSV at 24 h after infection was determined by RT-qPCR to assess the PRRSV N protein-encoding transcript levels. The experiment was repeated three times. The significant differences between the mAb-PN9cx3-treated-and-infected groups and the PRRSV-infected only group (0 μM) are marked by asterisks: * (*p* < 0.05) and ** (*p* < 0.01). **B** Viral yields in cell culture supernatants of PAMs infected with different PRRSV strains or infected with PRRSV preincubated with 1 μM mAb-PN9cx3. Supernatants were collected at 24 hpi and titrated in MARC-145 cells. All above-described experiments were repeated at least three times. The significant differences between infected PAMs of the 0 μM mAb-PN9cx3 group and 1 μM mAb-PN9cx3 group are marked by asterisks: * (*p* < 0.05). **C** Western blotting of PAMs infected with PRRSV-GD-HD and JXA1 virus at an MOI of 0.1 or PRRSVs pretreated (1-h incubation time) with 1 μM mAb-PN9cx3. After inoculation with a mAb-virus mixture, the PAMs were incubated for 1 h, and unbound PRRSV virions were removed by washing the cells with fresh medium. The PAMs were then incubated for 24 h at 37 °C before lysis for SDS-PAGE and Western blot analysis of the PRRSV N protein levels. **D** Western blotting of PAMs infected with VR2332 and NADC30-like HNhx virus at an MOI of 0.1 or with PRRSVs pretreated (1-h incubation time) with 1 μM mAb-PN9cx3. After inoculation with a mAb-virus mixture, the PAMs were incubated for 1 h, and unbound PRRSV virions were removed by washing the cells with fresh medium. The PAMs were then incubated for 24 h at 37 °C before lysis for SDS-PAGE and Western blotting to determine the PRRSV N protein levels.

**Figure 4:**
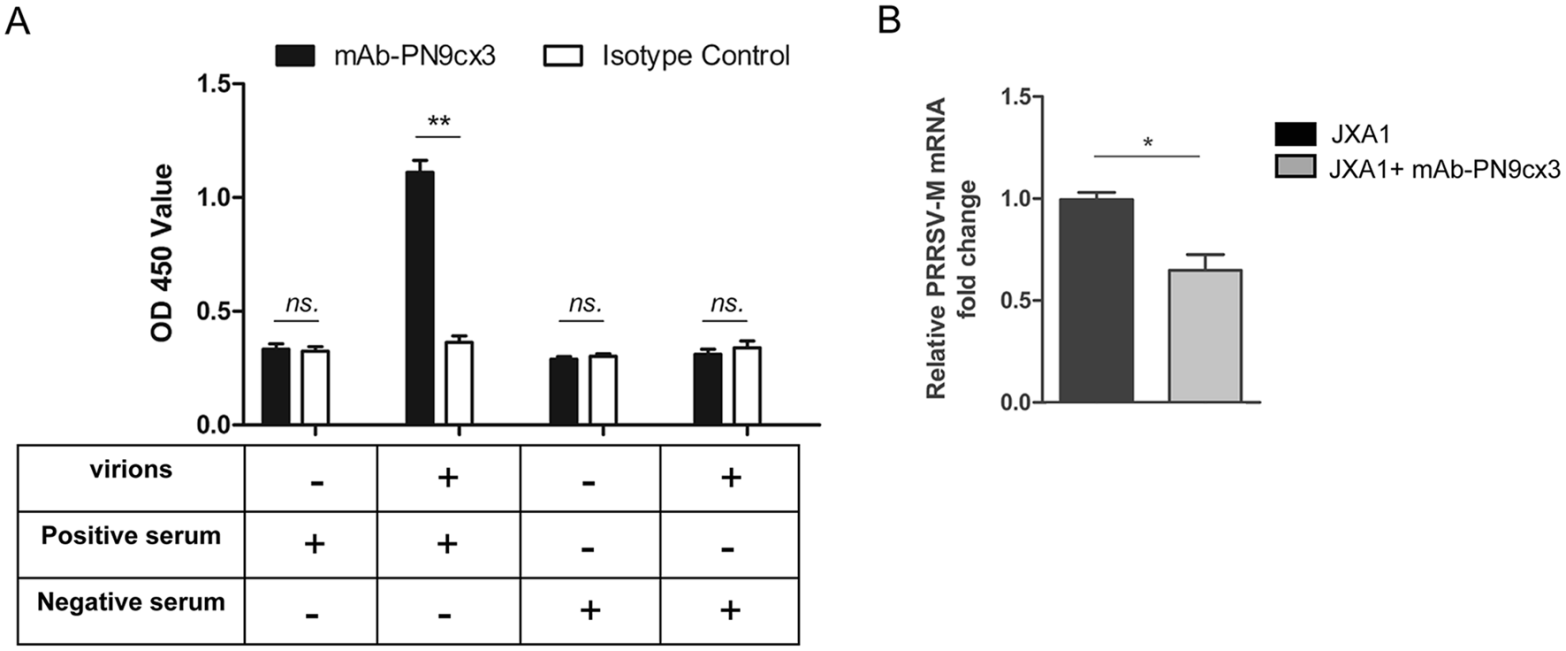
**mAb-PN9cx3 blocks the binding of PRRSV virions to PAMs**. **A** Sandwich ELISA for measuring the virion numbers in cell culture supernatants (100 μL) of PRRSV-infected MARC-145 cells (SD16 strain, 10^6.5^ TCID_50_/mL) or an equal volume of fresh DMEM containing 10% FBS. Either mAb-PN9cx3 or IgG isotype control (mAb-2G8) was used as the coating antibody for virion capture. PRRSV convalescent-phase swine serum (SD16 strain) or swine serum negative for anti-PRRSV antibodies was used for the detection of captured PRRSV virions. All the experiments were repeated at least three times. The significant differences between different groups are marked by asterisks: ** (*p* < 0.01). **B** mAb-PN9cx3 inhibited PRRSV virion attachment to PAMs. A mixture of PRRSV virions (SD16 strain) at an MOI of 0.1 and 1 μM mAb-PN9cx3 was incubated for 1 h at 37 °C, prechilled on ice and added to PAMs (1 × 10^6^), and the cells were then incubated for 2 h at 4 °C, washed three times with cooled PBS, and harvested for RT-qPCR analysis to determine the PRRSV-RNA levels. PAMs inoculated with virus alone (without mAb-PN9cx3) were included as a control. The experiment was repeated at least three times. The significant differences between two groups are marked by asterisks: * (*p* < 0.05).

The abovementioned IFA data demonstrated that mAb-PN9cx3 shows broad-spectrum recognition of heterogeneous isolates of both *PRRSV* genotypes as well as the ability to neutralize the infectivity of the PRRSV-SD16 strain. Because this HP-PRRSV isolate is distinct from the VR2385 strain used for hybridoma screening, we further investigated whether mAb-PN9cx3 could act as a broad neutralizing antibody against infection with both *PRRSV-1* and *PRRSV-2* in PAMs. To test this assumption, five representative PRRSV strains were selected: VR2332 (classical PRRSV strain), GD-HD (HP-PRRSV), JXA1 (HP-PRRSV), NADC30-like HNhx and GZ-11 (*PRRSV-1* Chinese isolate). Moreover, as partial-dose-dependent neutralization was observed in the above data, 1.0 μM mAb-PN9cx3 was selected because the neutralizing activity of 1.0 μM mAb-PN9cx3 against PRRSV-SD16 in PAMs appeared to be marginal, as evidenced by a 50% reduction in PRRSV mRNA and a more than 50% reduction in PRRSV-N protein in PAMs (Figures 2A and C). Mirroring results were obtained for PRRSV strain SD16; virus preincubation with 1.0 μM mAb-PN9cx3 sufficiently blocked the replication of all other tested strains (although relatively weak inhibition was observed for VR2332), as evidenced by decreased PRRSV-N RNA levels (Figure 3A), reduced numbers of progeny virions in the supernatants of infected PAMs (Figure 3B), and lower intracellular PRRSV-N protein levels (Figures 3C and D). The N protein level of GZ11 cannot be detected by mAb-6D10, and these data were thus not included in the WB results. Although only these five strains were tested in the neutralization assays, they represented the breadth of variation found among PRRSV isolates and shared at most only 60% nucleotide sequence identity. Notably, this spectrum of reactivity was similar to that observed with our previously described broad-spectrum IgM isotype neutralizing mAb mAb-PR5nf1 [26]. To further understand the mechanism through which mAb-PN9cx3 inhibited PRRSV replication, a virion capture assay based on sandwich ELISA was conducted, as shown in Figure 4A; in the ELISA, mAb-PN9cx3 served as the coating antigen (for virion capture), and probing was performed with PRRSV convalescent serum. The results showed a significant change in the OD value that was consistent with a direct interaction between mAb-PN9cx3 and PRRSV virions. Moreover, the incubation of PAMs with a PRRSV-mAb-PN9cx3 mixture at 4 °C (to avoid triggering endocytosis and subsequent virion internalization) significantly reduced the PRRSV-RNA levels, which represented the numbers of virions attached to the outer surfaces of PAMs (Figure 4B). Taken together, these findings demonstrated that mAb-PN9cx3 acted as a neutralizing anti-PRRSV mAb that blocked the attachment of virions of both PRRSV species to susceptible cells.

### mAb-PN9cx3 treatment significantly alleviates lung pathological lesions in piglets upon challenge with HP-PRRSV or NADC30-like PRRSV

**Figure 5:**
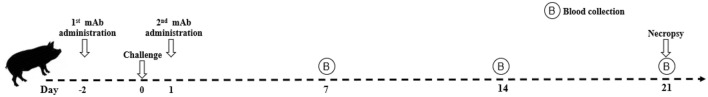
**Schematic illustration of the animal experiment protocol.**

mAbs of the IgG isotype are suitable for in vivo applications because the passive transfer of convalescent PRRSV serum to susceptible piglets protects the recipients from PRRS. Thus, we investigated whether the passive administration of mAb-PN9cx3 to piglets would confer broad-spectrum protection against challenge with heterogeneous PRRSV strains, as observed in vitro*,* particularly for the HP-PRRSV and NADC30-like PRRSV strains, which are currently the predominant PRRS outbreak strains in China. Such studies would be of special interest as protection against infection with NADC30-like PRRSV isolates has not been achieved using current MLVs because these strains exhibit the greatest degree of genetic variation relative to the *PRRSV-2* vaccine prototype strain VR2332. Therefore, the cross-protective efficiency of mAb-PN9cx3 against challenge with two typical strains representative of the currently prevalent strains circulating in China, HP-PRRSV-JXA1 and PRRSV NADC30-like strain HNhx, was evaluated in 4-week-old piglets. The details of the challenge protocol are illustrated in Figure 5.

**Figure 6:**
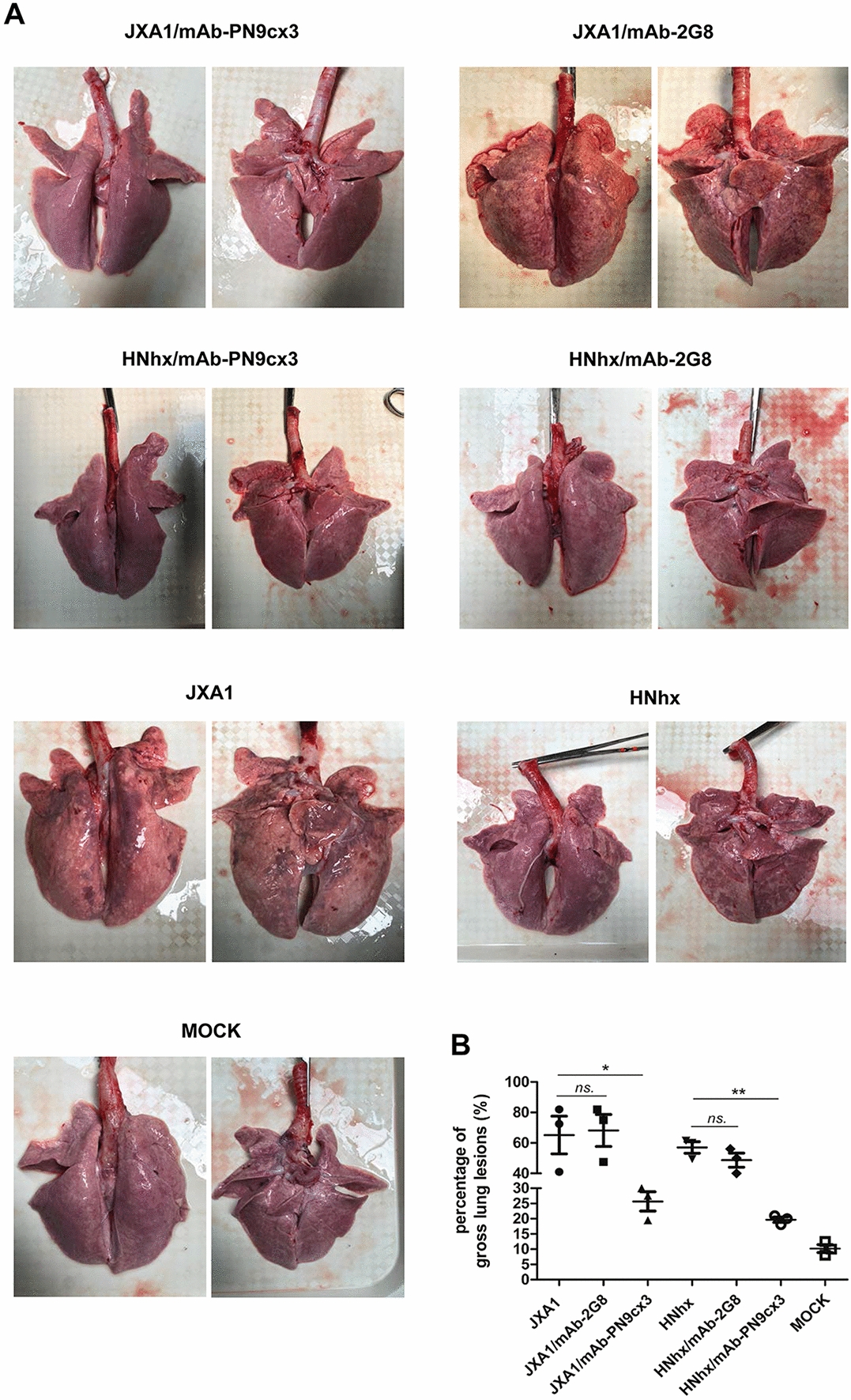
**mAb-PN9cx3 administration alleviates the lung pathological lesions observed after PRRSV challenge in vivo. A** A total of 28 piglets were randomly divided into seven groups (*n* = 4): one group served as the negative control (MOCK) group, the JXA1 and HNhx groups were challenged with HP-PRRSV-JXA1 and NADC30-like HNhx, respectively, the JXA1/mAb-PN9cx3 and HNhx/mAb-PN9cx3 groups were treated with mAb-PN9cx3 and challenged with HP-PRRSV-JXA1 and NADC30-like HNhx, respectively, and the JXA1/mAb-2G8 and HNhx/mAb-2G8 groups were administered the isotype mAb (2G8) and then challenged with HP-PRRSV-JXA1 and NADC30-like HNhx, respectively. For each group, representative images were captured immediately after piglets were autopsied at 21 dpi. **B** The gross pathological changes found in the animals of each group were quantified using a scoring (100-point) system. The significant differences between the mAb-PN9cx3-treated groups (JXA1/mAb-PN9cx3 and HNhx/mAb-PN9cx3) or the antibody-isotype control groups (JXA1/mAb -2G8 and HNhx/mAb-2G8) and the PRRSV-challenged groups (JXA1 and HNhx) are marked by asterisks: * (*p* < 0.05) and ** (*p* < 0.01).

After the administration of mAb-PN9cx3 or isotype control (mAb-2G8) as indicated followed by PRRSV challenge, the gross pathological changes in the lungs of all the piglets were recorded upon autopsy at 21 dpi. As demonstrated in Figure 6A, the lung lesions in the HP-PRRSV-JXA1 and NADC30-like HNhx challenge groups were phenotypically similar, and the challenge viruses resulted in extensive pneumonia and severe pathological changes (pulmonary oedema and white-coloured consolidations) (Figure 6A). To better quantify these pathological changes, a lung gross lesion score system was applied as previously described [33]. As shown in Figure 6B, the gross lung lesion scores of the piglets challenged with either PRRSV strain were significantly higher than the scores of the piglets in the MOCK control group, which did not exhibit gross lesions in the lungs. Therefore, the administration of mAb-PN9cx3 before and after virus challenge was effective and successfully protected the recipient piglets from HP-PRRSV-JXA1 and NADC30-like HNhx infection, as demonstrated by the marked alleviation of lung pathological lesions and the reduction in the lung gross lesion scores detected in the mAbs-PN9cx3-treated virus-challenged groups compared with the untreated virus-challenged groups (Figure 6B). In contrast, the treatment of piglets with isotype control mAb-2G8 before and after infection with either HP-PRRSV-JXA1 or NADC30-like HNhx strains had no protective effect, and similar lung lesion scores were obtained for both challenge groups (Figure 6B).

**Figure 7:**
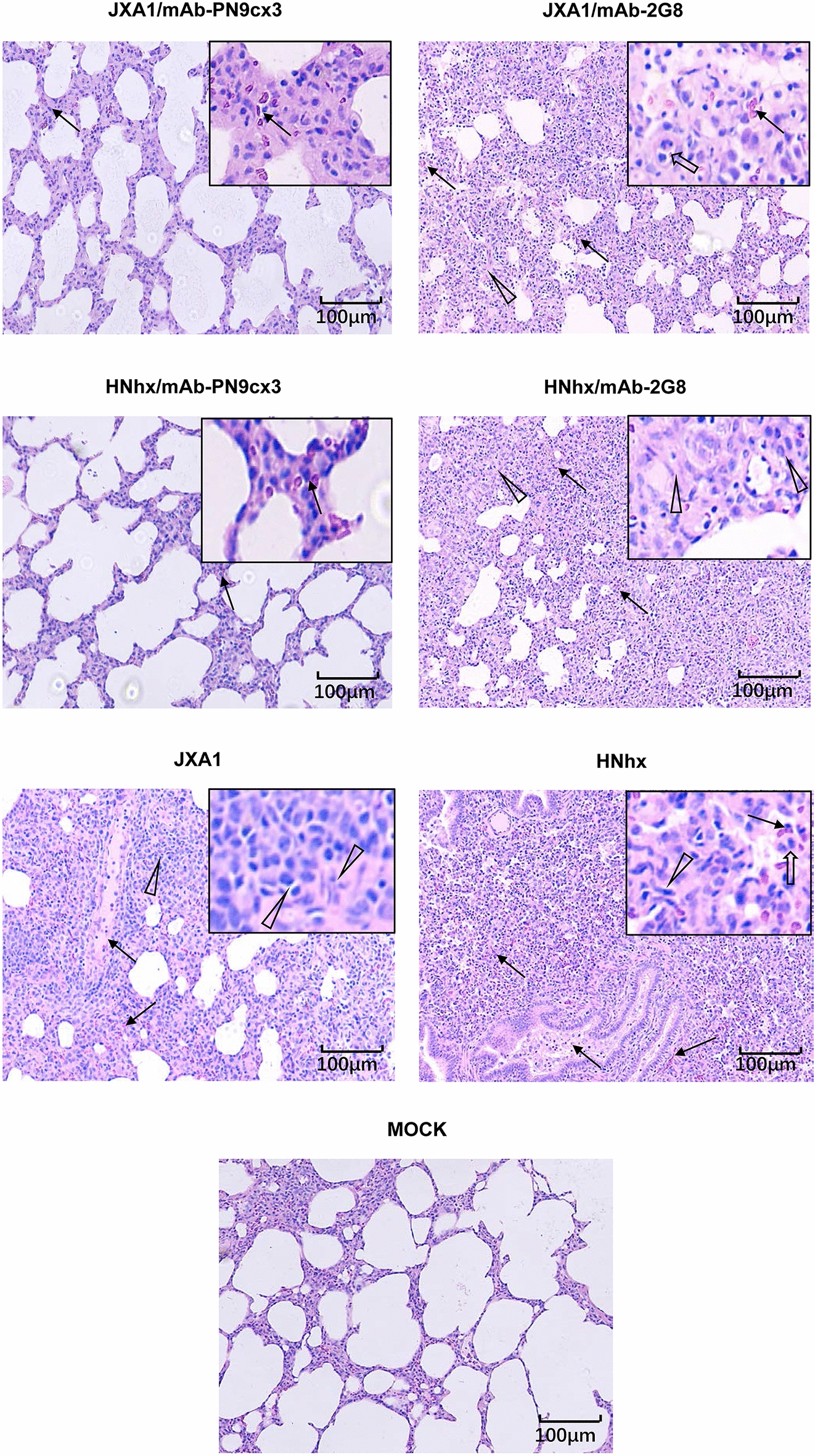
**mAb-PN9cx3 administration alleviates the lung pathological lesions induced by PRRSV challenge in vivo.** Tissue samples from the lungs of all the animals autopsied at 21 dpi were fixed in 10% neutral buffered formalin, embedded in paraffin blocks and sectioned for histological analysis using established accepted procedures. The sections were stained with haematoxylin and eosin (H&E) to facilitate the observation of micropathological changes. Representative images were captured, and the scale bar represents a length of 100 μm.

To better evaluate the protective efficacy of mAb-PN9cx3 against PRRSV, the microscopic pathological lesions in each piglet group were examined and compared. The HP-PRRSV-JXA1 challenge group exhibited classic interstitial pneumonia, with characteristic multifocal thickening within alveolar septa and spaces, the collapse of alveolar cells (distinct from type 2 pneumocyte hyperplasia associated with neutrophilic infiltration), and serous exudation and fibrosis within bronchioles or alveolar spaces (Figure 7). Moreover, the NADC30-like HNhx challenge group exhibited histopathological lesions similar to those observed in the tissues of HP-PRRSV-JXA1-challenged piglets and showed the most prominent type 2 pneumocyte hyperplasia signs among all the groups (Figure 7). No microscopic lesions were observed in the piglets of the MOCK group. The mAb-PN9cx3-treated piglets inoculated with either HP-PRRSV-JXA1 or NADC30-like HNhx exhibited comparable degrees of mild alveolar septal thickening that resembled the tissue changes found in the uninfected controls. Conversely, the parallel isotype control mAb-2G8-treated group showed severe histopathological lesions that were very similar in distribution and type to those found in the untreated PRRSV-challenge groups (Figure 7). Furthermore, the dynamics of the IV-injected mouse mAbs and swine anti-mouse antibody responses were evaluated. On the one hand, as demonstrated in Additional file 1A, the serum levels of mouse antibodies in the different groups showed rapid reduction from 7 to 14 dpi but were maintained at a similar level from 14 to 21 dpi. On the other hand, our data suggested that anti-mouse antibodies could be detected within 7 dpi (as determined by the evaluated OD value), and the OD value continually increased up to 14 dpi but then remained at a similar level from 14 to 21 dpi (Additional file 1B). Notably, anti-mouse antibodies in the mAb-PN9cx3-injected groups were also reactive with mAb-2G8, similar to the results found with mAb-PN9cx3, which suggested that the anti-mouse antibody mainly recognizes the constant region (Fc) of the mAb. Moreover, the virus neutralization activity of serum obtained from the different groups at 23 dpi was evaluated using the corresponding challenging viruses JXA1 (Additional file 2A) and HNhx (Additional file 2B). Consistent with the amount of mAb-PN9cx3 remaining in serum, a decreasing trend of infected cells was observed in the mAb-PN9cx3-treated piglets at the same fold-dilution (Additional file 2). Taken together, these data suggest that mAb-PN9cx3 provides efficient cross-protection against lung injury caused by infection with either HP-PRRSV-JXA1 or PRRSV NADC30-like HNhx strains in vivo*.*

### The administration of mAb-PN9cx3 effectively reduces the viral loads in PAMs and hilar lymph nodes of infected piglets

**Figure 8:**
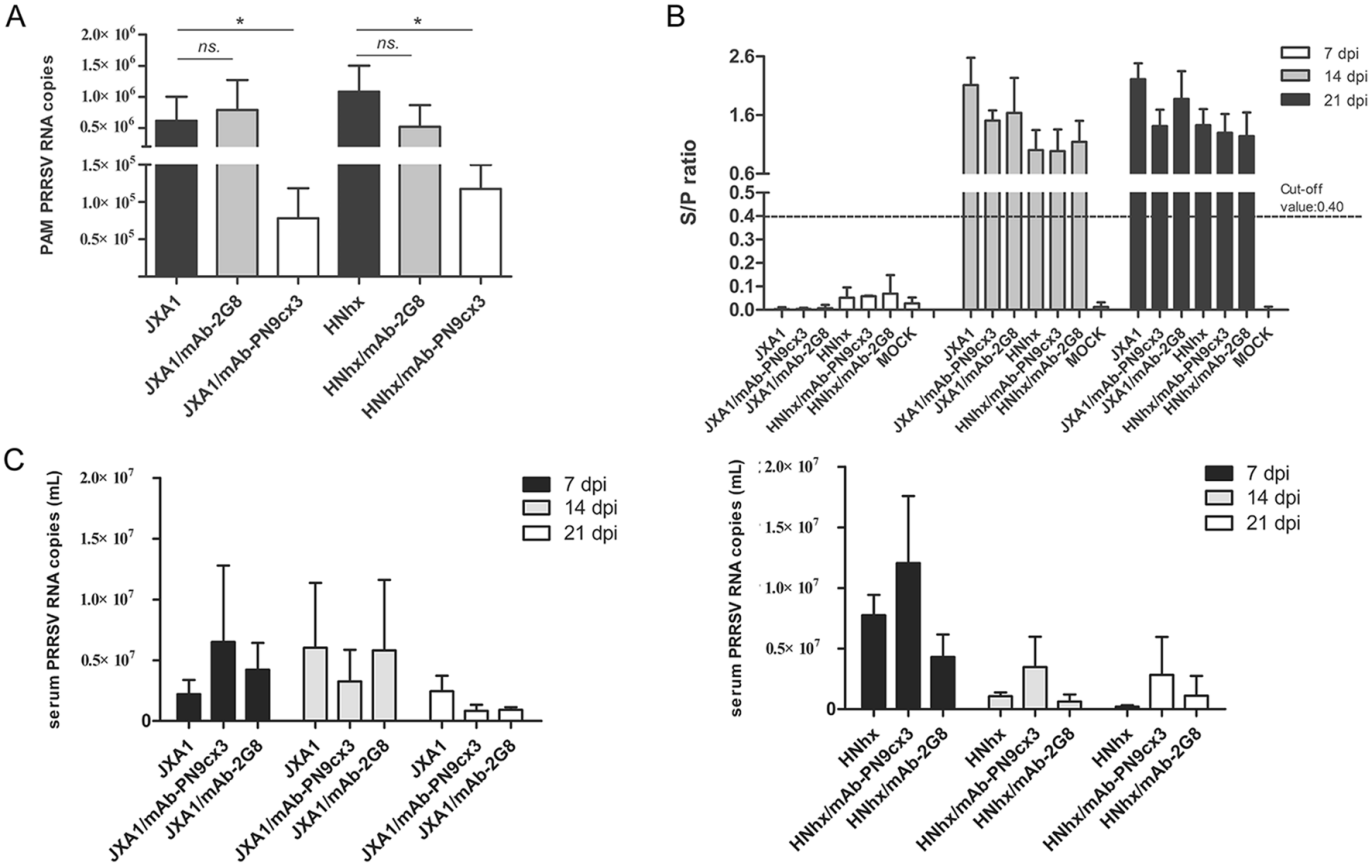
**mAb-PN9cx3 administration effectively reduces the viral loads in PAMs. A** A total of 1 × 10^7^ PAMs per animal were obtained from the treated piglets (JXA1/mAb-PN9cx3 and HNhx/mAb-PN9cx3) and the antibody-isotype control piglets (JXA1/mAb-2G8 and HNhx/mAb-2G8) during autopsy at 21 dpi and then harvested using the TRIzol reagent for reverse transcription. The significant differences in the results relative to those found for the PRRSV challenge groups (JXA1 and HNhx) are marked by asterisks: * (*p* < 0.05). **B** Serum samples from each animal were collected at 7, 14, and 21 dpi and subjected to an ELISA using an IDEXX HerdChek PRRS X3 ELISA kit to determine the occurrence of seroconversion after PRRSV inoculation. **C** Serum samples from each animal were collected at 7, 14, and 21 dpi and harvested using the TRIzol reagent for reverse transcription and qPCR analysis to determine the number of PRRSV-N gene copies in serum. No significant differences among the mAb-PN9cx3-treated groups (JXA1/mAb-PN9cx3 and HNhx/mAb-PN9cx3), antibody-isotype control groups (JXA1/mAb-2G8 and HNhx/mAb-2G8) and PRRSV-challenged groups (JXA1 and HNhx) were observed.

Because the abovementioned data demonstrated the protective efficacy of mAb-PN9cx3 in piglets against challenge with either virulent PRRSV strain, we further evaluated the degree of protection provided by mAb-PN9cx3 treatment by examining the viral loads in peripheral blood and cells of multiple organs, such as PAMs and pulmonary hilar lymph nodes (LNs), collected during necropsy at 21 dpi. As demonstrated in Figure 8A, the viral RNA levels (as determined by absolute quantification) in the PAM samples from the mAb-PN9cx3-treated groups were significantly reduced by 90% (one log_10_) compared with the levels found in the samples from the untreated virus-challenged and isotype mAb-treated control groups. These results align with the observed alleviation of lung pathological lesions in the mAb-PN9cx3-treated piglets. However, these data also suggest that the administration of mAb-PN9cx3 could not completely abolish PRRSV replication, as evidenced by the fact that all piglets challenged with PRRSV were seroconverted, as detected by ELISA by 14 dpi (Figure 8B). Moreover, the serum RNA levels were also evaluated, and the results revealed that the serum viral loads were comparable among the mAb-PN9cx3-treated, mAb-2G8-treated (isotype control), and PRRSV challenge groups at the same time points (regardless of the virus strain used for challenge) (Figure 8C). This observation was somehow conflicting because previous reports strongly suggest a correlation between the viremia level and the disease severity after PRRSV infection [40, 41]. This difference might be due to the high dose of the challenge virus (2 × 10^5^ TCID_50_) used in this study compared to the dose of 10^3^ to 10^4^ TCID_50_ of challenge virus used in other studies [41, 42]. It is possible that a shorter experiment time (3 weeks) might affect our data because a previous study suggested that the viremia peak in most HP-PRRSV-infected piglets can be observed between 3 and 4 weeks post-infection [43].

**Figure 9:**
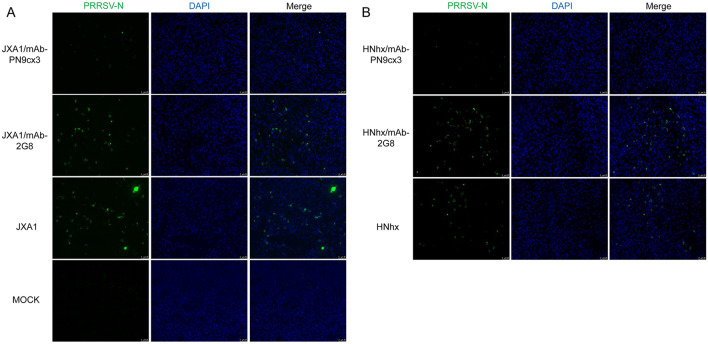
**mAb-PN9cx3 administration inhibits PRRSV replication in hilar lymph nodes. A** Hilar lymph nodes from the JXA1/mAb-PN9cx3 JXA1/mAb-2G8, JXA1 and MOCK groups were collected at necropsy and embedded in OCT compound in cryomoulds for cryostat sectioning into 5-μm-thick slices. The slices were mounted onto gelatine-coated slides, fixed with ice-cold acetone, and then air-dried. PRRSV antigen was detected using anti-PRRSV nucleocapsid (N) monoclonal antibody (mAb-6D10) and visualized using FITC-labelled goat anti-mouse secondary antibodies. **B** Hilar lymph nodes from the HNhx/mAb-PN9cx3 HNhx/mAb-2G8 and HNhx groups were collected at necropsy for cryostat sectioning followed by the detection of PRRSV antigen using anti-PRRSV nucleocapsid (N) monoclonal antibody (mAb-6D10). The results were visualized using FITC-labelled goat anti-mouse secondary antibodies.

To further evaluate the virus loads, the draining LNs (pulmonary hilar LNs) of piglets were cryosectioned and stained for PRRSV-N protein via immunofluorescence because viral replication in lymph organs is considered a sign of persistent PRRSV infection in vivo [44, 45]. Compared with the MOCK groups, which showed no detectable PRRSV-N antigen, the sectioned pulmonary hilar LN samples from PRRSV-challenged animals exhibited elevated numbers of PRRSV-infected cells (Figure 9A). Moreover, greater numbers of PRRSV-positive cells were observed in the HP-PRRSV-JXA1-challenge group than in the NADC30-like HNhx-challenge group. Nevertheless, mAb-PN9cx3 treatment provided all the recipients with markedly effective protection against infection with HP-PRRSV-JXA1 and NADC30-like HNhx, as demonstrated by the low number of PRRSV-positive cells observed in these sections. In contrast to the results obtained with mAb-PN9cx3 treatment, the mAb-2G8-treated groups exhibited a similar degree of PRRSV-positive cells in pulmonary hilar LNs as the untreated PRRSV-inoculated groups. Additionally, a slight increase in the number of PRRSV-positive cells was found in the mAb-2G8-treated group after challenge with NADC30-like HNhx compared with the untreated parallel NADC30-like HNhx-infected group (Figure 9B). Therefore, in addition to the prevention of PRRSV-induced lung injury, these results demonstrate that mAb-PN9cx3 provides efficient cross-protection against infection by both HP-PRRSV and NADC30-like PRRSV strains in vivo; this protection was reflected by significantly lower viral loads observed in both PAMs and draining LNs, and together, these findings suggest that mAb-PN9cx3 might also prevent persistent PRRSV infection.

### mAb-PN9cx3 treatment reverses the alterations in the expression profiles of certain genes induced by viral infection in PAMs from PRRSV-challenged piglets

**Figure 10:**
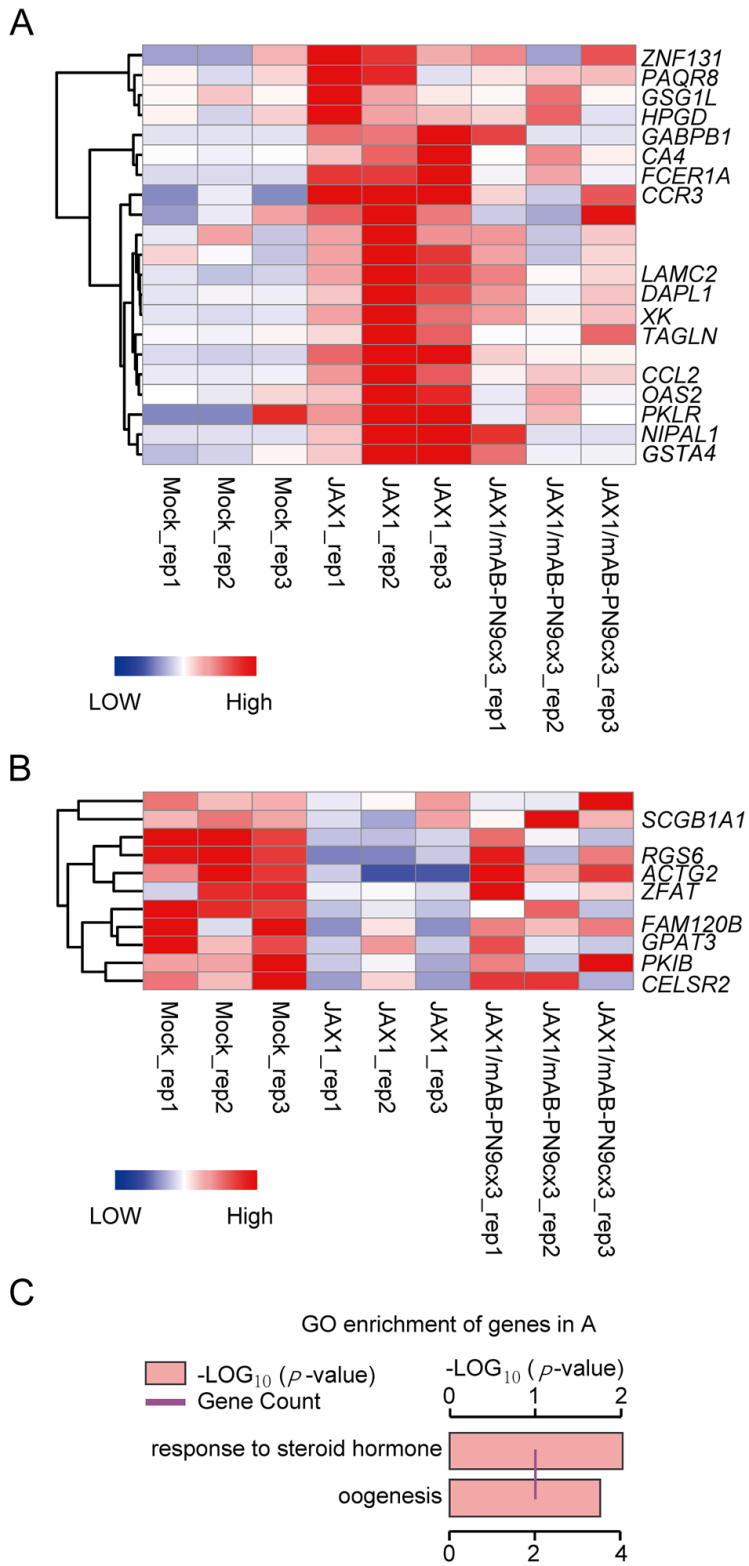
**Transcriptome profiling and gene ontology (GO) enrichment analyses of PAMs from PRRSV-JXA1-infected pigs with or without mAb-PN9cx3 treatment. A** Heatmap analysis of genes upregulated by PRRSV-JXA1 in infected PAMs but that exhibited reversal of this upregulation in piglets treated with mAb-PN9cx3 before and after infection. **B** Heatmap analysis showing genes downregulated by PRRSV-JXA1 in infected PAMs but that exhibited reversal of this downregulation in piglets treated with mAb-PN9cx3 before and after infection. The expression levels are indicated by the colour key: blue corresponds to low-level expression, and red corresponds to high-level expression. The row order reflects the hierarchical clustering results, and the values are scaled in the row direction. **C** Gene ontology (GO) enrichment analysis of genes upregulated in PAMs induced with PRRSV-JXA1 but that exhibited reversal of this upregulation in piglets treated with mAb-PN9cx3 before and after infection.

To better understand the mechanisms underlying the protective effects of mAb-PN9cx3 against PRRSV in vivo, PAM samples from three piglets randomly selected from each group (except for the antibody-isotype control group) were subjected to RNA sequencing followed by global transcriptome analysis to understand the transcription profiles relevant to viral infection and protection from infection. Although previous transcriptome profiling has revealed that hundreds of genes show altered expression after PRRSV infection, little is known about the specific genes involved in PRRSV pathogenesis in vivo or whether distinct PRRSV strain-specific transcriptome profiles exist among highly heterogeneous PRRSV isolates. In this study, mAb-PN9cx3 treatment protected piglets from PRRSV-induced pathogenesis, even though the replication of PRRSV in host cells was not completely blocked. Subsequently, we screened for genes that exhibited infection-induced alterations in expression and that showed expression levels in mAb-PN9cx3-treated piglets that were similar to those found in the MOCK-infected group. Based on the aforementioned methods used to generate these preliminary results, the transcriptome profiles of PAMs were compared, and the results revealed that hundreds of genes were either upregulated or downregulated in the PRRSV-infected groups (data not shown). Unexpectedly, only 21 genes were upregulated by PRRSV-JXA1 infection and exhibited expression levels that reverted to levels comparable to those found in MOCK control group after mAb-PN9cx3 treatment (designated JXA1-upregulated-mAb-PN9cx3-reversed in Additional file 3) (Figure 10A). Concurrently, only 11 genes were downregulated by PRRSV-JXA1 infection and showed a reversal of this downregulation by mAb-PN9cx3 treatment (designated JXA1-downregulated-mAb-PN9cx3-reversed in Additional file 4) (Figure 10B). These data suggest that most genes exhibiting an alteration in expression induced by PRRSV-JXA1 infection in vivo were unrelated to PRRSV pathogenesis; this conclusion is also supported by the fact that the disease phenotype changed substantially after mAb-PN9cx3 treatment, even though the host genes or pathways involved in HP-PRRSV-JXA1 pathogenesis were limited. Notably, with the exception of unannotated genes excluded from the analysis, the GO enrichment analysis assigned functional terms for genes with infection-induced altered expression that included the “response to steroid hormone” and “oogenesis” pathways (Figure 10C, Additional file 5).

Importantly, although a cytokine storm is considered as the major contributing factor underlying HP-PRRSV pathogenesis, no genes involved in cytokine signalling or proinflammatory responses were identified and enriched (Figures 10A and B). On the one hand, certain genes involved in the chemokine axis were identified within the set of JXA1-upregulated mAb-PN9cx3-reversed genes, including CCR3 and CCL2 (Figure 10A). Thus, activation of the chemokine axis likely contributed more to HP-PRRSV pathogenesis than cytokine signalling. Notably, FCER1A, the Fc fragment of IgE receptor Ia that binds to the Fc region of IgE and is responsible for initiating the allergic response, was upregulated by HP-PRRSV-JXA1 infection. On the other hand, GO enrichment analysis of the JXA1-downregulated mAb-PN9cx3-reversed genes did not identify an enriched pathway, but SCGB1A1 (Secretoglobin Family 1A Member 1) was downregulated after HP-PRRSV-JXA1 infection (Figure 10B).

**Figure 11:**
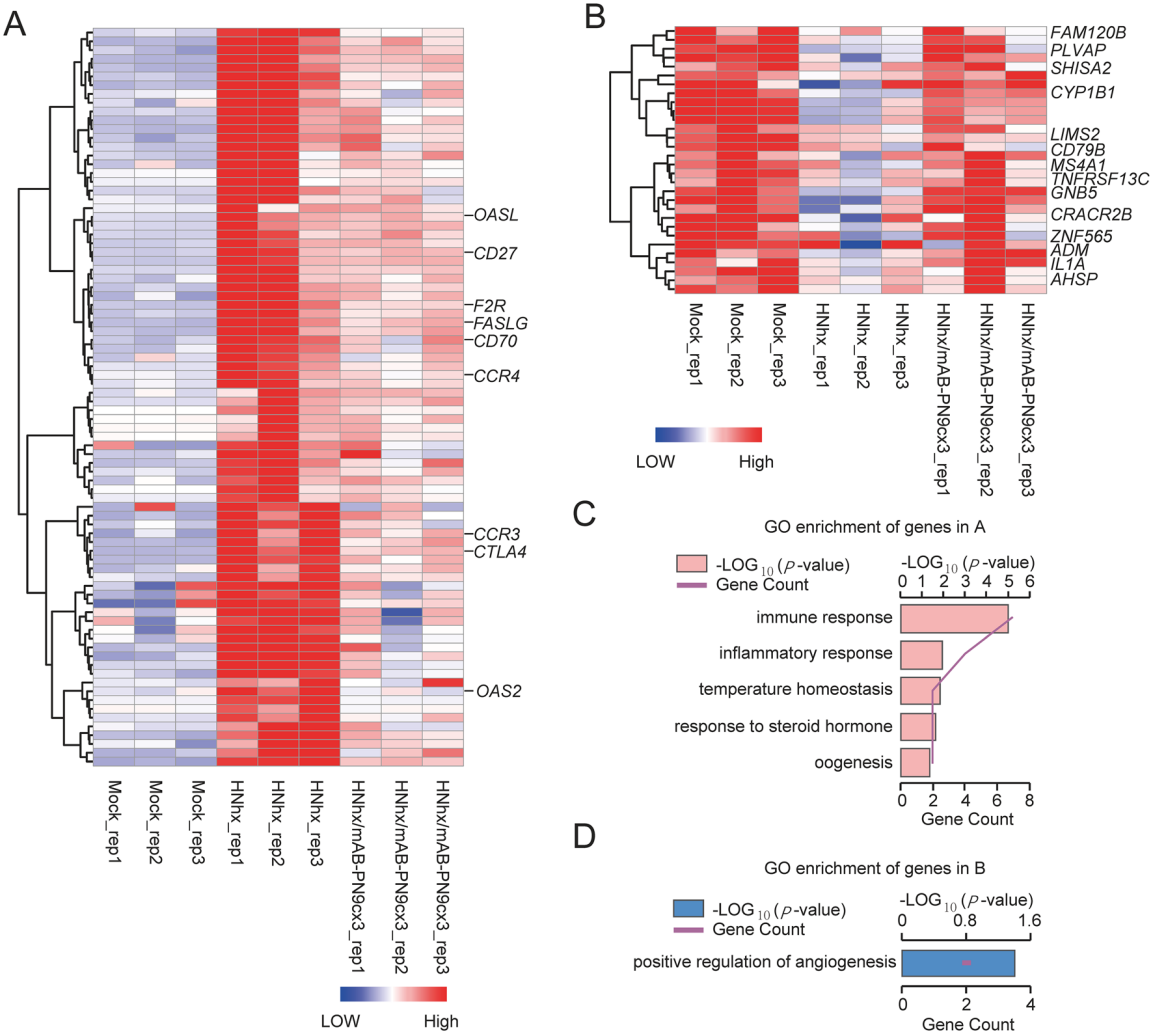
**Transcriptome profiling and GO enrichment analysis of expressed genes in PAMs from PRRSV-NADC30 like-HNhx-infected pigs with or without mAb-PN9cx3 treatment**. **A** Heatmap analysis of genes upregulated by PRRSV-NADC30 like-HNhx infection but that exhibited reversal of this upregulation in PAMs of piglets treated with mAb-PN9cx3 before and after infection. **B** Heatmap analysis of genes downregulated by PRRSV-NADC30-like HNhx infection but that exhibited reversal of this downregulation in PAMs of mAb-PN9cx3-treated-and-infected piglets. The expression levels are indicated using a colour key: blue corresponds to low-level expression, and red corresponds to high-level expression. The row order reflects the hierarchical clustering results, and the values are scaled in the row direction. **C** Gene ontology (GO) enrichment analysis of genes upregulated by PRRSV-NADC30-like HNhx infection but that exhibited reversal of this upregulation in PAMs of piglets treated with mAb-PN9cx3 before and after infection. **D** Gene ontology (GO) enrichment analysis of genes downregulated by PRRSV-NADC30-like HNhx infection but that exhibited reversal of this downregulation in PAMs of piglets treated with mAb-PN9cx3 before and after infection.

Conversely, the analysis of the PRRSV-NADC30-like HNhx-infected and mAb-PN9cx3-treated challenge groups revealed that 84 and 30 genes were upregulated and downregulated by HNhx infection, respectively, and showed expression levels after mAb-PN9cx3 treatment that were similar to the MOCK levels (Figures 11A and B and Additional files 6 and 7). With the exception of unannotated genes, the GO enrichment analysis of the functional terms assigned to the genes showing altered expression pinpointed several pathways involved in the host-PRRSV interaction (Figures 11C and D and Additional files 8 and 9), such as the immune and inflammatory response and temperature homeostasis pathways, which were designated HNhx-upregulated-mAb-PN9cx3-reversed pathways. Intriguingly, except for genes related to the chemokine axis, several negative regulators of immune responses were identified, such as CTLA-4 [46], CD70, and CD27 [47] (Figure 11A), and CTLA-4 and CD27 have been identified as T cell-specific genes with roles as costimulatory molecules. Although further research is needed to confirm their expression in PAMs of swine species, it is possible that the bronchoalveolar lavage contains lymphocytes penetrated below alveoli during PRRSV infection and that the downregulation of these costimulatory molecules occurred in these lymphocytes rather than PAMs. Moreover, it is worth noting that PAQR8 (Membrane Progestin Receptor Beta) and its corresponding pathways were enriched, and “response to steroid hormone” and “oogenesis” were identified as both JXA1/HNhx-upregulated-mAb-PN9cx3-reversed genes and pathways (Figure 10C and Figure 11C). Although the exact role played by PAQR8 in PRRSV pathogenesis is unclear, its known natural role in inhibiting both oestradiol- and progesterone-associated innate and cellular immune responses [48, 49] suggests that PAQR8 and its corresponding pathways negatively regulate anti-PRRSV immunity in vivo.

In addition to HNhx-upregulated mAb-PN9cx3-reversed genes and their corresponding enriched pathways, 30 genes were identified as a HNhx-downregulated mAb-PN9cx3-reversed gene cluster comprised mainly of nonannotated genes. In fact, no genes and pathways in this gene cluster were found to be enriched with the exception of ADM and IL1A (enriched as “positive regulation of angiogenesis”) (Figure 11D). However, we noticed that CYP1B1 (Cytochrome P450, Family 1, Subfamily B, Polypeptide 1), which plays a key role in oestrogen metabolism [50], was downregulated after HNhx infection, and this result was consistent with the upregulation of progestin receptor PAQR8 (Figure 11D), which potentially contributes to the negative regulation of anti-PRRSV immunity in vivo as well. Moreover, certain B cell-specific markers, such as MS4A1, TNFRSF13C, and CD79B, were downregulated, which might be a consequence of lymphocyte penetration into alveoli, similar to the downregulated T cell-specific genes described above. Collectively, the abovementioned data suggest that mAb-PN9cx3 treatment reverses the PRRSV infection-induced alterations in gene expression observed in vivo. It appears that genes within the chemokine axis and pathways such as the response to steroid hormone were upregulated by infection with both HP-PRRSV and NADC30-like HNhx isolates. Moreover, it seems that NADC30-like HNhx infection particularly altered the expression of genes involved in the negative regulation of host immune responses to ultimately enhance PRRSV pathogenesis.

In conclusion, our data demonstrated that an in vitro-tested broad neutralizing monoclonal antibody could confer broad in vivo protection to piglets when administered before and after challenge with heterogeneous virulent PRRSV isolates. Moreover, mAb-PN9cx3 administration also reversed the PRRSV-induced alterations in gene expression in PAMs, a natural target cell of PRRSV infection. Furthermore, transcriptome profiling of PAMs pinpointed a potential role of the chemokine axis and steroid hormone pathways in PRRSV pathogenesis in vivo.

## Discussion

Antibody responses against PRRSV were initially thought to be an ineffective component of the PRRSV-protective immune response and could even be deleterious due to concerns of antibody-dependent enhancement effects observed in vitro [51]. However, this view has changed because neutralizing antibodies are now viewed as key players in protection against PRRSV [14, 52]. Unfortunately, the high degree of observed variability among PRRSV isolates suggests that both *PRRSV-1* and *PRRSV-2* strains are constantly evolving to adapt to the existing host immunity by spawning new variants that continuously cause new outbreaks [53]. Therefore, the creation of an effective vaccine to target the constantly evolving PRRSV is daunting.

In recent years, broad neutralizing antibodies have been reported in swine herds exposed to cycling PRRSV field strains [24, 54]. Although an attempt to neutralize porcine mAbs against PRRSV has been performed [55], a single mAb with the ability to neutralize both *PRRSV-1* and *PRRSV-2* was not reported until recently. Our previous study identified mAb-PR5nf1 as an IgM isotype mAb with broad neutralizing activity against both *PRRSV-1* and *PRRSV-2* [26]. Using mAb-PR5nf1 as a reference mAb, we identified the IgG isotype mAb-PN9cx3 in this study and found that it also exhibited broad neutralizing activity. Although the highest concentration of mAb-PN9cx3 used in the in vitro assessment of the virus-neutralizing ability of the antibody was 2 μM, which is higher than the concentration of mAb-PR5nf1 (0.4 μM) needed to achieve comparable results [26], the total amounts of mAb-PN9cx3 and mAb-PR5nf1 (approximately 400 μg in mass) applied in the neutralizing assays were similar, which implies that the pentameric structure of IgM might block the entry of virions into susceptible cells better than IgG, and this finding is consistent with previous research demonstrating that a recombinant IgM antibody targeting influenza B blocks viral infection with greater breadth and potency than an antibody with the IgG isotype [56]. Moreover, a previous study investigating broad neutralizing human mAbs against influenza B virus demonstrated that the minimum dose required for the 50% inhibition of influenza B virus varied from less than 1 μg to 100 μg/mL (approximately 0.66 μM for the average molecular weight of human IgG) depending on the clone of mAbs and the viral strain used in the assay [57]. Therefore, the dose of mAb-PN9cx3 used in our study for PRRSV neutralization was higher than that used for influenza B virus but is still within an accepted range.

Because in vivo experiments have suggested that neutralizing antibodies (Nabs) account for the humoural immunity associated with vaccine-induced protection, passive-transfer experiments have been conducted, and the results have demonstrated that the administration of sufficient amounts of Nabs can protect piglets from PRRSV challenge [25, 58, 59]. Consequently, the injection of PRRSV-specific neutralizing mAbs for the prevention of PRRS should be a feasible approach for preventing disease but has never been reported. Compared with IgM, which does not readily traverse vascular walls to enter infected tissues, IgG is expected to be more suitable for in vivo application. Moreover, the results of an ongoing clinical trial of recombinant human mAb (MEDI8897) developed for the prophylaxis of RSV disease in infants support the prophylactic application of mAb against PRRSV [60]. Therefore, two IV injections that together contained 20 mg of mAb-PN9cx3 were administered to piglets based on the medium dose (25 mg) used in the MEDI8897 human trial. Currently, the cDNA of both the light chain and heavy chain of mAb-PN9cx3 has been sequenced and cloned into mammalian expression plasmids (data not shown). Our preliminary data suggested that the recombinantly expressed mAb-PN9cx3 from 293f cells cotransfected with plasmids encoding the light and heavy chains could be successfully assembled and maintains the same reactivity of purified mAb-PN9cx3 from hybridoma to PRRSV-infected MARC-145 cells (data not shown). The large-scale production of recombinant mAb-PN9cx3 could be a practical and cost-effective strategy for the prophylactic prevention of PRRSV in swine herds.

Based on our in vivo data, the administration of two 10-mg mAb-PN9cx3 inoculations separated by a 3-day interval prophylactically prevented piglets from severe lung injury after PRRSV challenge regardless of the viral strain; this result was consistent with the viral neutralizing activity observed for mAb-PN9cx3 against PRRSV in vitro. More importantly, mAb-PN9cx3 provided a significantly broader range of protection against heterogeneous PRRSV strains, as demonstrated by observed protection against pathology induced by challenge with HP-PRRSV-JXA1 and PRRSV-NADC30-like HNhx strains and a superior degree of cross-protection than that provided by MLV vaccinations. Mechanistically, mAb-PN9cx3 blocked the binding of PRRSV to cells, which prevented viral infection in vivo, and this finding was consistent with the in vitro observations of significant inhibition of virion attachment to permissive PAMs after preincubation of the virus with mAb-PN9cx3. In vivo, a higher than 90% reduction in the PRRSV-RNA levels was observed in PAMs from mAb-PN9cx3-treated PRRSV-infected piglets compared with PAMs from untreated PRRSV-challenged piglets. Moreover, because persistent viral replication in lymph organs is considered the major factor underlying persistent PRRSV infection [44, 45], only trace levels of PRRSV antigen were detected in hilar lymph node tissues from mAb-PN9cx3-treated PRRSV-infected piglets, which indicates that mAb-PN9cx3 could also potentially prevent persistent PRRSV infection.

Previous in vivo studies comparing the pathogenesis of a highly pathogenic PRRSV strain, a classical PRRSV strain, and an attenuated PRRSV vaccine strain demonstrated that rapid replication of HP-PRRSV in pigs could trigger a cytokine storm characterized by sustained expression of proinflammatory cytokines and chemokines, and this cytokine storm would then trigger a robust inflammatory response leading to high mortality rates [61–63]. However, little is known about the early events responsible for triggering a cytokine storm. Therefore, we conducted transcriptome profiling of PAMs isolated from experimentally infected piglet groups to identify genes whose expression was altered by PRRSV infection but which showed a reversal of this change in response to mAb treatment. Unexpectedly, we found that the expression profiles of only a small number of genes associated with PRRSV pathogenicity showed this reversal, and only a few of these genes were linked to proinflammatory cytokines; however, certain alternate genes, such as SCGB1A1, have been demonstrated to be a major innate sensor that restricts respiratory syncytial virus (RSV) infection by mediating neutrophilic inflammation and mucosal IFN production [64]. On the one hand, it is notable that pathways involved in the response to steroid hormone were upregulated in PAMs of piglets challenged with either HP-PRRSV or NADC30-like HNhx isolate. After considering the natural role played by steroid hormone pathways (including oestrogen and progesterone) in the negative regulation of immune responses, it is most likely that PRRSV pathogenesis relies on multiple pathways to achieve immune inhibition. On the other hand, in NADC30-like strain HNhx-infected piglets, several negative regulators of immune responses were also upregulated and showed a reversal of this upregulation in response to mAb treatment, which suggests that the infection of PAMs with the PRRSV-NADC30 like strain HNhx led to stronger and more comprehensive inhibition of more host immune response pathways.

For a long time, the antigenic variability among PRRSV isolates has hampered the development of effective prevention and control strategies based on antibody-mediated virus neutralization [65]. In fact, the major neutralization targets among PRRSV antigens remain controversial. Although it has been postulated that GP5, the major glycosylated envelope protein encoded by PRRSV-ORF5, acts as a major inducer of NAbs, the immunization of pigs with recombinant GP5 leads to enhanced clinical disease following experimental challenge [66]. More recently, neutralizing epitopes located within minor envelope proteins such as GP2, 3, and 4 have been frequently reported for both *PRRSV-1* and *PRRSV-2* isolates [25, 67–69]. Therefore, the traditional view of the GP5-M heterodimer as a major neutralization target of anti-PRRSV NAbs has been frequently challenged in recent years [54, 69, 70]. Nevertheless, our understanding of PRRSV envelope antigens and epitopes related to viral neutralization remains insufficient due to the lack of crystal structure information for all PRRSV envelope proteins, which hinders our ability to understand the structural basis of virus-receptor interactions and antibody-mediated neutralization.

In our previous investigation of PRRSV broad neutralizing mAb-PR5nf1, although mAb-PR5nf11 was able to capture PRRSV virions from culture supernatants of virus-infected cells, mAb-PR5nf1 did not bind to GP5 overexpressed in 293 T cells or SF9 cells. Therefore, it appears that the epitope recognized by mAb-PR5nf1 is unlikely to be located in PRRSV-GP5 but is rather located on the surfaces of viral particles [26]. Moreover, the generation of such an epitope also depends on an intact viral envelope structure and requires post-translational modifications within a functional Golgi apparatus, and PNGase F treatment (glycosylation) of the PRRSV virion could interfere with the binding of mAb-PR5nf11 to viral particles, as evidenced by the reduced OD value obtained in sandwich ELISA using mAb-PR5nf11 as the capture antibody [26]. Mirroring mAb-PR5nf1, mAb-PN9cx3 is also capable of capturing viral particles but cannot recognize GP3 expressed in HEK-293 T cells or in baculovirus-infected SF9 cells (data not shown). It is possible that mAb-PN9cx3 recognizes a glycosylated conformational epitope similarly to mAb-PR5nf11, but this hypothesis requires further investigation. In addition, we also analysed the sequence similarity of GP3 proteins encoded by the PRRSV strains tested in this investigation (Table 3 and Additional file 10) and demonstrated that the sequence similarity of GP3 from these strains ranged from 57.7% to 98.4; therefore, it is unlikely that the sequence similarity of GP3 is a major determinant for mAb-PN9cx3 specificity. Nevertheless, the data obtained in this study suggest the existence of a conserved epitope within PRRSV virions and that the broad neutralizing activity against diverse PRRSV strains conferred by mAb-PR5nf1 (IgM) is not a consequence of IgM polyreactivity. However, the exact epitope recognized by mAb-PN9cx3 remains elusive and thus warrants future investigations. We are currently working on the purification of PRRSV virions for use in complex docking experiments with mAb-PN9cx3 for cryo-electron microscopy (cryo-EM) to reveal the structural basis of the mAb-PN9cx3-dependent neutralization of PRRSV.

**Table 3:** **Sequence comparison of GP3 among different PRRSV strains (× 100%.)**

	GZ11	HNhx	VR2332	GD-HD	JXA1	SD16
SD16	57.7	79.5	86.6	98.4	97.6	1
JXA1	57.7	79.9	86.2	98.4	1	97.6
GD-HD	57.7	80.3	87	1	98.4	98.4
VR2332	59.7	82.3	1	87	86.2	86.6
HNhx	59.3	1	82.3	80.3	79.9	79.5
GZ11	1	59.3	59.7	57.7	57.7	57.7

In conclusion, our results demonstrate that the PRRSV-specific broad neutralizing mAb PN9cx3 is a promising antiviral agent for providing prophylactic protection against PRRSV infection in piglets. In addition, mAb-PN9cx3 might provide broad protection against highly distinct *PRRSV-2* NADC30-like isolates in vivo. Our research also provides proof-of-concept evidence showing that mAb-based therapy or prophylaxis is feasible in swine, although further optimization is required. Thus, the treatment of piglets with mAb-PN9cx3 represents a novel method for the future control and prevention of PRRSV.

## Supplementary Information

**Additional file 1:** **Evaluation of mAb-PN9cx3 in the serum of piglets and anti-mouse antibody responses in piglets injected with mAb-PN9cx3.** **A.** The piglet serum samples from the indicated groups were diluted in PBS at a 1:5 ratio and then subjected to ELISA of mouse IgG using a mouse IgG quantification ELISA kit. PBS-diluted pig serum from the MOCK group supplemented with purified mAb-PN9cx3 was used for standard curve calculation. **B.** For evaluation of the anti-antibody response in mAb-treated piglets, 96-well polystyrene microplates were coated with 200 ng of mAb-PN9cx3 or mAb-2G8 and blocked with 5% skim milk. Diluted pig serum samples (1- to 256-fold dilution in PBS) from the indicated groups were added to evaluate the generation of swine anti-mouse IgG. Serum samples from piglets without mAb treatment were included as controls. The significant differences between the indicated groups are marked by asterisks: * (*p* < 0.05), ** (*p* < 0.01), and *** (*p* < 0.001).

**Additional file 2: Evaluation of PRRSV neutralizing activity in serum samples from piglets. A**. Serum samples from the PRRSV-JXA1-infected group, negative control group (MOCK) and mAb-PN9cx3-treated group (JXA1/mAb-PN9cx3) at 21 dpi were gradient diluted in DMEM and incubated with PRRSV-JXA1 at an MOI of 0.1 for 1 h at 37 °C prior to the inoculation of MARC-145 cells. An IFA with the PRRSV-N-specific mAb-PP7EF11 was conducted 18 h after inoculation, and the florescence spots were counted and compared to those obtained with the negative control group. The significant differences between the indicated groups are marked by asterisks: * (*p* < 0.05) and ** (*p* < 0.01). The term “ns” refers to “no sense”. **B**. Serum samples from the PRRSV-HNhx-infected group, negative control group (MOCK) and mAb-PN9cx3-treated group (HNhx/mAb-PN9cx3) at 21 dpi were gradient diluted in DMEM and incubated with PRRSV-HNhx virus at an MOI of 0.1 for 1 h at 37 °C prior to the inoculation of MARC-145 cells. An IFA with the PRRSV-N-specific mAb-PP7EF11 was conducted 18 h after inoculation, and the florescence spots were counted and compared with those obtained with the negative control group. The significant differences between the indicated groups are marked by asterisks: * (*p* < 0.05) and ** (*p* < 0.01). The term “ns” represents “no sense”.

**Additional file 3.** **Summary of PRRSV-JXA1-upregulated-mAb-PN9cx3-reversed genes.** The transcript abundances were converted to transcripts per million (TPM) units, and rep1, 2 and 3 represent data from three piglets in the MOCK, JXA1 and JXA1/mAb-PN9cx3 groups.

**Additional file 4.** **Summary of PRRSV-JXA1-downregulated-mAb-PN9cx3-reversed genes.** The transcript abundances were converted to transcripts per million (TPM) units, and rep1, 2 and 3 represent data from three piglets in the MOCK, JXA1 and JXA1/mAb-PN9cx3 groups.

**Additional file 5.** **GO enrichment of PRRSV-JXA1-upregulated-mAb-PN9cx3-reversed genes.** The GO terms enriched in PRRSV-HNhx-upregulated-mAb-PN9cx3-reversed pig genes are collected in Additional file 6. Sheets 1 and 2 show the enriched biological process (BP)- and molecular function (MF)-associated GO terms, respectively.

**Additional file 6.** Summary of PRRSV-HNhx-upregulated mAb-PN9cx3-reversed genes. The transcript abundances were converted to transcripts per million (TPM) units, and rep1, 2 and 3 represent data from three piglets in the MOCK, HNhx and HNhx/mAb-PN9cx3 groups.

**Additional file 7.** **Summary of PRRSV-JXA1-downregulated mAb-PN9cx3-reversed genes.**The transcript abundances were converted to transcripts per million (TPM) units, and rep1, 2 and 3 represent data from three piglets in the MOCK, HNhx and HNhx/mAb-PN9cx3 groups.

**Additional file 8.** GO enrichment of PRRSV-HNhx-upregulated-mAb-PN9cx3-reversed genes. The GO terms and KEGG pathways enriched in PRRSV-HNhx-upregulated-mAb-PN9cx3-reversed pig genes are collected in Additional file 9. Sheets 1, 2, and 3 show the enriched biological process (BP)-, cellular component (CC)-, and molecular function (MF)-associated GO terms, respectively. Sheet 4 shows the enriched KEGG pathways.

**Additional file 9.** **GO enrichment of PRRSV-HNhx-downregulated-mAb-PN9cx3-reversed genes.** The GO terms and KEGG pathways enriched in PRRSV-HNhx-downregulated-mAb-PN9cx3-reversed pig genes are collected in Additional file 10. Sheet 1 shows the enriched biological process (BP)-associated GO terms. Sheet 2 shows the enriched KEGG pathways.

**Additional file 10.** **Phylogenetic tree of the GP3 protein sequences of different PRRSV strains.**

## Data Availability

The RNA-seq datasets generated in this study can be found in the aforementioned NCBI Gene Expression Omnibus (GEO Accession no. GSE156504).

## Abbreviations

PRRSV: Porcine reproductive and respiratory syndrome virus
ORFs: Open reading frames
MLV: Modified live virus
HP-PRRSV: Highly pathogenic PRRSV
NAbs: Neutralizing antibodies
mAbs: Monoclonal antibodies
PAMs: Porcine alveolar macrophages
SDS-PAGE: Sodium dodecyl sulphate–polyacrylamide gel electrophoresis
qPCR: Quantitative real-time PCR
LNs: Lymph nodes
BALF: Bronchoalveolar lavage fluid
